# Involvement of Cerebellar Neural Circuits in Active Avoidance Conditioning in Zebrafish

**DOI:** 10.1523/ENEURO.0507-20.2021

**Published:** 2021-06-02

**Authors:** Wataru Koyama, Ryo Hosomi, Koji Matsuda, Koichi Kawakami, Masahiko Hibi, Takashi Shimizu

**Affiliations:** 1 Division of Biological Science, Graduate School of Science, Nagoya University, Nagoya, Aichi 464-8602, Japan; 2 Laboratory of Molecular and Developmental Biology, National Institute of Genetics, and Department of Genetics, Graduate University for Advanced Studies (SOKENDAI), Mishima, Shizuoka 411-8540, Japan

**Keywords:** cerebellum, active avoidance, operant conditioning, botulinum toxin, nitroreductase, zebrafish

## Abstract

When animals repeatedly receive a combination of neutral conditional stimulus (CS) and aversive unconditional stimulus (US), they learn the relationship between CS and US, and show conditioned fear responses after CS. They show passive responses such as freezing or panic movements (classical or Pavlovian fear conditioning), or active behavioral responses to avoid aversive stimuli (active avoidance). Previous studies suggested the roles of the cerebellum in classical fear conditioning but it remains elusive whether the cerebellum is involved in active avoidance conditioning. In this study, we analyzed the roles of cerebellar neural circuits during active avoidance in adult zebrafish. When pairs of CS (light) and US (electric shock) were administered to wild-type zebrafish, about half of them displayed active avoidance. The expression of botulinum toxin, which inhibits the release of neurotransmitters, in cerebellar granule cells (GCs) or Purkinje cells (PCs) did not affect conditioning-independent swimming behaviors, but did inhibit active avoidance conditioning. Nitroreductase (NTR)-mediated ablation of PCs in adult zebrafish also impaired active avoidance. Furthermore, the inhibited transmission of GCs or PCs resulted in reduced fear-conditioned Pavlovian fear responses. Our findings suggest that the zebrafish cerebellum plays an active role in active avoidance conditioning.

## Significance Statement

An animal can associate a neutral conditioned stimulus and an aversive unconditioned stimulus, and escape to avoid an aversive stimulus. This is called active avoidance conditioning and is essential for an animal’s survival. Although the amygdala and habenula nucleus are reportedly involved in active avoidance conditioning, the roles of other brain regions are largely unknown. We describe the roles of the cerebellum during active avoidance in adult zebrafish. The neurotoxin botulinum toxin-mediated inhibition of granule cells (GCs) or Purkinje cells (PCs), or the ablation of PCs, suppressed active avoidance conditioning. Our findings indicate that the cerebellum plays a positive role in active avoidance conditioning.

## Introduction

An animal can associate two environmental stimuli and consequently shows certain behaviors. Broadly, there are neutral cues or conditional stimuli (CSs), such as sound or light, and aversive (noxious) cues or unconditional stimuli (USs), such as an electric shock. If an animal receives repeated pairs of CSs and USs, it associates both to show fear responses on exposure to CSs. Consequently, CS-dependent aversive effects are expected. The animal may show two types of fear responses, “passive” and “active,” on exposure to a CS. Passive responses are known as “classical fear conditioning” or “Pavlovian fear conditioning.” This includes motor responses, including freezing behavior and panic movements (Sacchetti et al., 2002; Agetsuma et al., 2010; Amo et al., 2014; Lal et al., 2018), and autonomic reactions such as CS-evoked bradycardia responses (Supple and Leaton, 1990; Supple and Kapp, 1993; Supple et al., 1993; Yoshida and Hirano, 2010; Kotajima et al., 2014; Matsuda et al., 2017). It also shows active responses that result in the avoidance of noxious stimuli. Conditioning that elicits active (voluntary) responses is known as operant conditioning, and a type of operant conditioning that induces behaviors to avoid noxious stimuli is called “active avoidance” (Skinner, 1984). The amygdala in mammals is involved in both classical fear conditioning and active avoidance conditioning (Duvarci and Pare, 2014; Herry and Johansen, 2014). In zebrafish, a region in the dorsal telencephalon that is considered to be equivalent to the amygdala (Dm), and the habenula-raphe neural circuits, are reportedly involved in adaptive active avoidance conditioning (Aoki et al., 2013; Amo et al., 2014; Lal et al., 2018). However, it is still largely unknown if other neural circuits, including the cerebellum, are involved in fear conditioning.

The cerebellum plays important roles in some forms of motor coordination and motor learning, and is also involved in higher cognitive emotional functions (Ito, 2005, 2006, 2008). The functions of the cerebellum rely on neural circuits that are conserved among vertebrates (Hashimoto and Hibi, 2012; Hibi et al., 2017). Purkinje cells (PCs) and granule cells (GCs) are major GABAergic and glutamatergic neurons in the cerebellum, and receive two inputs from outside of the cerebellum. PCs receive climbing fibers (CFs), which are axons from the inferior olivary nuclei (IOs). GCs receive mossy fibers (MFs) from precerebellar nuclei located in various regions of the brain. MF information is conveyed by GC axons, called parallel fibers (PFs). PCs integrate the two inputs and send outputs to the outside of the cerebellum through efferent neurons, which are deep cerebellar nuclei in mammals and eurydendroid cells in teleosts. The cerebellum of teleosts, including zebrafish, can be divided into the rostro-medial domain [valvula cerebelli (Va) and corpus cerebelli (CCe)] and the caudo-lateral domain [lobus caudalis cerebelli (LCa) and eminentia granularis (EG)] that are composed of different neural circuit structures (for review, see Hibi and Shimizu, 2012; Hibi et al., 2017). Cerebellar neural circuits are involved in classical conditioning, such as eye-blink conditioning. In mammals, lesions of the cerebellar vermis or IOs impair the acquisition of fear-conditioned bradycardia (Supple and Leaton, 1990; Kotajima et al., 2014). Inhibition of the vermis or efferent neurons with tetrodotoxin disrupts the consolidation of conditioned freezing responses in rats (Sacchetti et al., 2002). Lesions or chemical inhibition of the cerebellum also impair fear-conditioned bradycardia responses (Yoshida et al., 2004; Yoshida and Hirano, 2010). These studies suggest that the cerebellum is involved in classical fear conditioning. However, it is uncertain whether the cerebellar neural circuits control active avoidance conditioning.

Previous studies revealed that zebrafish can acquire classical fear conditioning and active avoidance conditioning from larval stages, although the timing to acquire robust conditioned behaviors varies depending on the experimental conditions (Aizenberg and Schuman, 2011; Valente et al., 2012; Matsuda et al., 2017; Lin et al., 2020). Zebrafish reliably show classical fear-conditioned bradycardia responses from the late larval stage [∼20 d postfertilization (dpf); Matsuda et al., 2017]. Cerebellar neurons in the CCe are activated during conditioned behaviors and inhibition of the GC transmission prolonged recovery from the conditioned responses (Matsuda et al., 2017), implying a role of the cerebellar neural circuits in classical fear conditioning in zebrafish. In operant conditioning, cerebellar neurons are also activated, and lesions in the cerebellum delay decision-making (Lin et al., 2020). However, specific inhibition of cerebellar neurons in active avoidance conditioning has not been reported. In this study, we transgenically expressed botulinum toxin (BoTx), a neurotoxin that inhibits neurotransmitter release, in GCs or PCs, or nitroreductase (NTR), which can convert a prodrug metronidazole (MTZ) to a cytotoxin (Pisharath et al., 2007; Tabor et al., 2014), in PCs, to study the roles of cerebellar neural circuits in active avoidance conditioning in adult zebrafish.

## Materials and Methods

### Ethics statement

The animal experiments in this study were approved by the Institutional Animal Experiment Committee and were conducted in accordance with the Regulations on Animal Experiments at the institute.

### Zebrafish

Wild-type zebrafish with the Oregon AB genetic background and two previously reported transgenic (Tg) lines, *gSA2AzGFF152B* (Takeuchi et al., 2015), which expresses a modified version of Gal4-VP16 (GAL4FF, also referred to as GFF) in the corpus cerebelli GCs, and *Tg(UAS:BoTxBLC-GFP)^icm21^*, which expresses the light chain of BoTx in a GAL4-dependent manner (Sternberg et al., 2016), were used. To generate the *Tg(aldoca:BoTxBLC-GFP)* line, the 5-kbp *aldolase Ca* (*aldoca*) promoter (Tanabe et al., 2010), the BoTxBLC-GFP gene (Sternberg et al., 2016), and the polyadenylation site (pAS) of pCS2+ were subcloned to the Tol2 vector pT2KDest-RfaF (Nojima et al., 2010) by Gateway (Thermo Fisher Scientific). To generate the *Tg(cbln12:Gal4FF)* line, the 2-kbp *cerebellin12* (*cbln12*) promoter (Dohaku et al., 2019), *GAL4FF* (Asakawa et al., 2008), and pAS of pCS2+ were subcloned to the Tol2 vector pT2ALR-Dest (Dohaku et al., 2019) by the Gateway system. To generate the *Tg(aldoca:NTR-TagRFPT)* line, the *aldoca* promoter, the epNTR-TagRFPT gene encoding a fusion protein of zebrafish codon-optimized enhanced-potency NTR (epNTR) and TagRFPT (Tabor et al., 2014), and pAS of pCS2+ were subcloned to pT2ARL-Dest by the Gateway system. To establish the Tg lines, 25 pg of the Tol2 plasmid and 25 pg of transposase-capped RNA were injected into one-cell-stage embryos. To inhibit GC transmission, *gSA2AzGFF152B* and *Tg(cbln12:Gal4FF)* were crossed with *Tg(UAS:BoTxBLC-GFP)*. Tg adult fish that harbored both *GAL4FF* and *GFP* were identified before conditioning by genotyping with primer pairs: 5′-AAGTGCGCCAAGTGTCTGAAGAAC-3′ (F) and 5′-GACCTGGACATGCTGCCTGCTGAT-3′ (R) for *GAL4FF*, and 5′-CGAACATAGCTAGCGTGACCGTGA-3′ (F) and 5′-TGGAGCACGTGTATCAGCTCATGC-3′ (R) for *BoTxBLC*. Those that did not harbor *GAL4FF* or *GFP* were used as control fish. Similarly, adult *Tg(aldoca:BoTxBLC-GFP)* fish were identified by BoTxBLC genotyping. Adult fish that did not have the transgene were used as control fish. Fish were maintained in a 14/10 h light/dark cycle (light 9 A.M. to 11 P.M.; dark 11 P.M. to 9 A.M.) at 28.5°C. Four- to 12-month-old adult fish were used in this study. All experiments were conducted without distinction between males and females.

### MTZ treatment

Adult *Tg(aldoca:NTR-TagRFPT)* fish (8–11 months old) were treated with 10 mm MTZ solution for 18 h and returned to their tank. Eleven days after treatment, the fish were subjected to behavior tests.

### Immunostaining and measurement

Cryosections 14 μm thick were prepared according to the previous publication (Bae et al., 2009). The sections were immunostained as previously described (Bae et al., 2009; Kani et al., 2010). The following antibodies were used: anti-GFP (1:1000, rat, Nacalai Tesque, catalog #04404-84, RRID: AB 10013361 or 1:1000, rabbit, MBL International, catalog #598, RRID: AB_591816) for BoTxBLC-GFP, anti-Neurod1 (1:400, mouse, ascites; Kani et al., 2010), and anti-parvalbumin 7 (1:1000, mouse monoclonal, ascites; Bae et al., 2009). The following secondary antibodies were used: Alexa Fluor 488 goat anti-rat (H + L, Invitrogen, Thermo Fisher Scientific, catalog #A11006, RRID: AB_2534074), CF488A anti-rabbit (H + L, Biotium Inc., catalog #20019, RRID: AB_10583180), and Alexa Fluor 568 goat anti-mouse IgG (H + L, Invitrogen, catalog #A11031, RRID: AB_144696). An LSM700 confocal laser-scanning microscope was used to obtain fluorescence images. GFP^+^ areas in the granular layer (GL) were measured by ImageJ software (https://imagej.nih.gov/ij/) (Table 1).

**Table 1:** Tg lines and expression of BoTxBLC-GFP

Tg lines	Expression	Sample number	Lateral sections (%)	Medial sections (%)	LCa (%)
*gSA2AzGFF152B;* *Tg(UAS:BoTxBLC-GFP)*	GCs (CCe>> LCa, EG)	1	14.7	69.5	0.2
		2	13.7	63.9	0.9
		3	6.4	48.6	0.2
*Tg(cbln12:Gal4FF);* *Tg(UAS:BoTxBLC-GFP)*	GCs (CCe, LCa, EG, TL)	1	38.0	35.6	44.2
		2	37.7	42.1	55.0
		3	55.6	50.9	50.5
*Tg(aldoca:BoTxBLC-GFP)*	PCs	1	96.4	97.7	NA
		2	95.9	96.8	
		3	100	99.4	

Sagittal sections from three *gSA2AzGFF152B;Tg(UAS:BoTxBLC-GFP)* or *Tg(cbln12:Gal4FF);Tg(UAS:BoTxBLC-GFP)* adult fish were stained with anti-GFP and anti-Neurod1 antibodies. Sagittal sections from three *Tg(aldcoa:BoTxBLC-GFP)* adult fish were stained with anti-GFP and anti-Pvalb7 antibodies. Two typical lateral and medial sections from each fish were used. For the GCs, the percentage of the GFP^+^ area in the GL area was determined by using ImageJ software. For the PCs, the number of GFP^+^ and Pvalb7^+^ cells was counted manually. The percentage of GFP^+^ cells to Pvalb7^+^ cells was determined. CCe, corpus cerebelli; EG, eminentia granularis; GC, granule cells; LCa, lobus caudalis cerebelli; NA, not applicable; TL, torus longitudinalis.

### Swimming performance test

Swimming performance was analyzed as previously described (Matsuda et al., 2017). Freely swimming adult fish were recorded by a CMOS camera (30 frames per second; fps). The head position of each fish was tracked using the Tracker (http://physlets.org/tracker) program. The distance and direction of head movements between two consecutive frames were calculated in Microsoft Excel. An event showing >90° in the direction change of two consecutive movements was counted as one turn. Average swimming speed and turning frequencies were calculated in Microsoft Excel.

### Active avoidance conditioning, Pavlovian fear conditioning, and response to electric shocks

Active avoidance conditioning was conducted by using a previously reported apparatus (Lal et al., 2018) and protocol (Aoki et al., 2013). Fish were maintained in a tank covered by white paper on the day before conditioning to prevent them from receiving special visual cues. For the conditioning, a white opaque tank (L41 cm × W17 cm × H12 cm) with transparent walls at both ends and a trapezoidal wedge (L10–20 cm × W17 cm × H5 cm) in the center of the tank was used. For the Pavlovian fear conditioning, a side compartment of the tank for active avoidance conditioning was used (L15.5 cm × W17 cm × H12 cm). Green LEDs (3.3-V DC, 2 A) and a pair of platinum mesh electrodes (12-V AC: 0.71 V/cm, 60 Hz) were used to provide CS and US, respectively. Behaviors were monitored by a CMOS camera (30 fps). The timing of CS and US was controlled with a DAQ interface (USB-6008; National Instruments Co) and laboratory-made software written in LabVIEW (National Instruments Co). Responses to electric shocks were examined by measuring swimming speed for 2 s before and after 10-s electric shocks after habituation for 20 min.

### Statistics

Data were analyzed and graphs were generated using GraphPad Prism (version 5.1) or the R software package (4.0.3; https://www.r-project.org/). Data are presented as the average **±** SEM. Statistical tests were applied as indicated in the figure legends. Additional statistical details are provided in Table 2.

**Table 2:** Summary of statistical analyses

Figure	Measurement	Type of test	Comparison	Statistical value
2*G*	Number of turns during free swimming	Welch’s *t* test	Learner WT vs non-learner WT	*p *=* *0.5924 *t*_(41)_ = 0.5397
2*H*	Swimming speed during free swimming	Welch’s *t* test	Learner WT vs non-learner WT	*p *=* *0.4405 *t*_(41)_ = 0.7791
2*J*	Swimming speed before and after electric shock	Welch’s *t* test	Before US vs after US in WT	*p *=* *0.002975 *t*_(12)_ = −4.539
3*A*	Number of turns during free swimming	Welch’s *t* test	Control vs 152B::BoTx	*p *=* *0.6224 *t*_(86)_ = −0.4942
3*B*	Swimming speed during free swimming	Welch’s *t* test	Control vs 152B::BoTx	*p *=* *0.7603 *t*_(86)_ = −0.3062
3*C*	Number of turns during free swimming	Welch’s *t* test	Control vs cbln12::BoTx	*p *=* *0.03522 *t*_(75)_ = −2.145
3*D*	Swimming speed during free swimming	Welch’s *t* test	Control vs cbln12::BoTx	*p *=* *0.3511 *t*_(75)_ = −0.9397
3*E*	Swimming speed before and after electric shock	Welch’s *t* test	Before US vs after US in 152B::BoTx	*p *=* *4.043e-05 *t*_(12)_ = −6.314
3*F*	Swimming speed before and after electric shock	Welch’s *t* test	Before US vs after US in cbln12::BoTx	*p *=* *0.01427 *t*_(12)_ = −3.130
3*G*	Learning rate of active avoidance	Fisher’s exact test	Control vs 152B::BoTx	*p *=* *5.574e-08
3*H*	Learning rate of active avoidance	Fisher’s exact test	Control vs cbln12::BoTx	*p *=* *0.01080
4*A*	Number of turns during free swimming	Welch’s *t* test	Control vs aldoca:BoTx	*p *=* *0.2164 *t*_(78)_ = 1.246
4*B*	Swimming speed during free swimming	Welch’s *t* test	Control vs aldoca:BoTx	*p *=* *0.6001 *t*_(78)_ = −0.5264
4*C*	Swimming speed before and after electric shock	Welch’s *t* test	before US vs after US in aldoca:BoTx	*p *=* *0.0001143 *t*_(12)_ = −6.248
4*D*	Learning rate of active avoidance	Fisher’s exact test	Control vs aldoca:BoTx	*p *=* *0.002116
4*E*	Number of trials in training session 1	Welch’s *t* test	Control vs aldoca:BoTx	*p *=* *0.3255 *t*_(20)_ = −1.031
4*E*	Number of trials in training session 2	Welch’s *t* test	Control vs aldoca:BoTx	*p *=* *0.7545 *t*_(20)_ = 0.3182
4*E*	Number of trials in training session 3	Welch’s *t* test	Control vs aldoca:BoTx	*p *=* *0.3865 *t*_(20)_ = 0.8856
4*E*	Number of trials in test session	Welch’s *t* test	Control vs aldoca:BoTx	*p *=* *0.2301 *t*_(20)_ = −1.360
5*M*	Number of turns during free swimming	Welch’s *t* test	Control vs aldoca:NTR	*p *=* *0.2728 *t*_(54)_ = −1.143
5*N*	Swimming speed during free swimming	Welch’s *t* test	Control vs aldoca:NTR	*p *=* *0.02462 *t*_(54)_ = 2.509
5*O*	Swimming speed before and after electric shock	Welch’s *t* test	Before US vs after US in aldoca:NTR	*p *=* *0.0003567 *t*_(12)_ = −6.371
5*P*	Swimming speed before and after electric shock	One-way ANOVA		*F*_(18,621)_ = 2.88 *p *=* *0.03943
5*P*	Swimming speed before and after electric shock	One-way ANOVA with Tukey’s *post hoc* test	WT vs 152B::BoTx	*p* = 0.6201
5*P*	Swimming speed before and after electric shock	One-way ANOVA with Tukey’s *post hoc* test	WT vs cbln12::BoTx	*p* = 0.08734
5*P*	Swimming speed before and after electric shock	One-way ANOVA with Tukey’s *post hoc* test	WT vs aldoca:BoTx	*p* = 0.7949
5*P*	Swimming speed before and after electric shock	One-way ANOVA with Tukey’s *post hoc* test	WT vs aldoca:BoTx	*p* = 0.9959
5*Q*	Learning rate of active avoidance	Fisher’s exact test	Control vs aldoca:NTR	*p *=* *0.008326
6*B*	Change of speed in training session	Two-way repeated measures ANOVA lines × trials interaction		*F*_(18,621)_ = 3.515 *p *=* *1.398e-06
6*B*	Change of speed in training session	Two-way repeated measures ANOVA lines factor		*F*_(2,69)_ = 19.92 *p *=* *1.483e-07
6*B*	Change of speed in training session	Two-way repeated measures ANOVA trials factor		*F*_(9,621)_ = 5.725 *p *=* *1.189e-07
6*B*	Change of speed in training session	Two-way repeated measures ANOVA with Tukey’s *post hoc* test	WT vs 152B::BoTx in trial 6	*p *=* *0.03243
6*B*	Change of speed in training session	Two-way repeated measures ANOVA with Tukey’s *post hoc* test	WT vs 152B::BoT in trial 7	*p *=* *0.04779
6*B*	Change of speed in training session	Two-way repeated measures ANOVA with Tukey’s *post hoc* test	WT vs 152B::BoT in trial 8	*p *=* *4.1e-09
6*B*	Change of speed in training session	Two-way repeated measures ANOVA with Tukey’s *post hoc* test	WT vs aldoca:BoTx in trial 8	*p *=* *2.6e-05
6*B*	Change of speed in training session	Two-way repeated measures ANOVA with Tukey’s *post hoc* test	WT vs 152B::BoTx in trial 9	*p *=* *0.005936
6*B*	Change of speed in test session	Two-way repeated measures ANOVA lines × trials interaction		*F*_(18,621)_ = 1.083 *p *=* *0.3649
6*B*	Change of speed in test session	Two-way repeated measures ANOVA lines factor		*F*_(2,69)_ = 28.35 *p *=* *1.398e-06
6*B*	Change of speed in test session	Two-way repeated measures ANOVA trials factor		*F*_(9,621)_ = 1.431 *p *=* *0.171
6*B*	Change of speed in training session	Two-way repeated measures ANOVA with Tukey’s *post hoc* test	WT vs 152B::BoTx	*p *<* *1e-22
6*B*	Change of speed in training session	One-way ANOVA with Tukey’s *post hoc* test	WT vs aldoca:BoTx	*p *<* *1e-22
6*B*	Change of speed in training session	One-way ANOVA with Tukey’s *post hoc* test	aldoca:BoTx vs 152B::BoTx	*p *=* *0.9425
6*C*	Learning rate of Pavlovian fear conditioning	Fisher’s exact test with BH *post hoc* test	WT vs 152B::BoTx	*p *=* *0.02892
6*C*	Learning rate of Pavlovian fear onditioning	Fisher’s exact test with BH *post hoc* test	WT vs aldoca:BoTx	*p *=* *0.02892
6*C*	Learning rate of Pavlovian fear conditioning	Fisher’s exact test with BH *post hoc* test	152B::BoTx vs aldoca:BoTx	*p *=* *1.000

In all figures, the data distribution was normal. WT, wild-type.

## Results

### Establishment of Tg zebrafish expressing botulinum toxin in GCs or PCs

**Figure 1.**
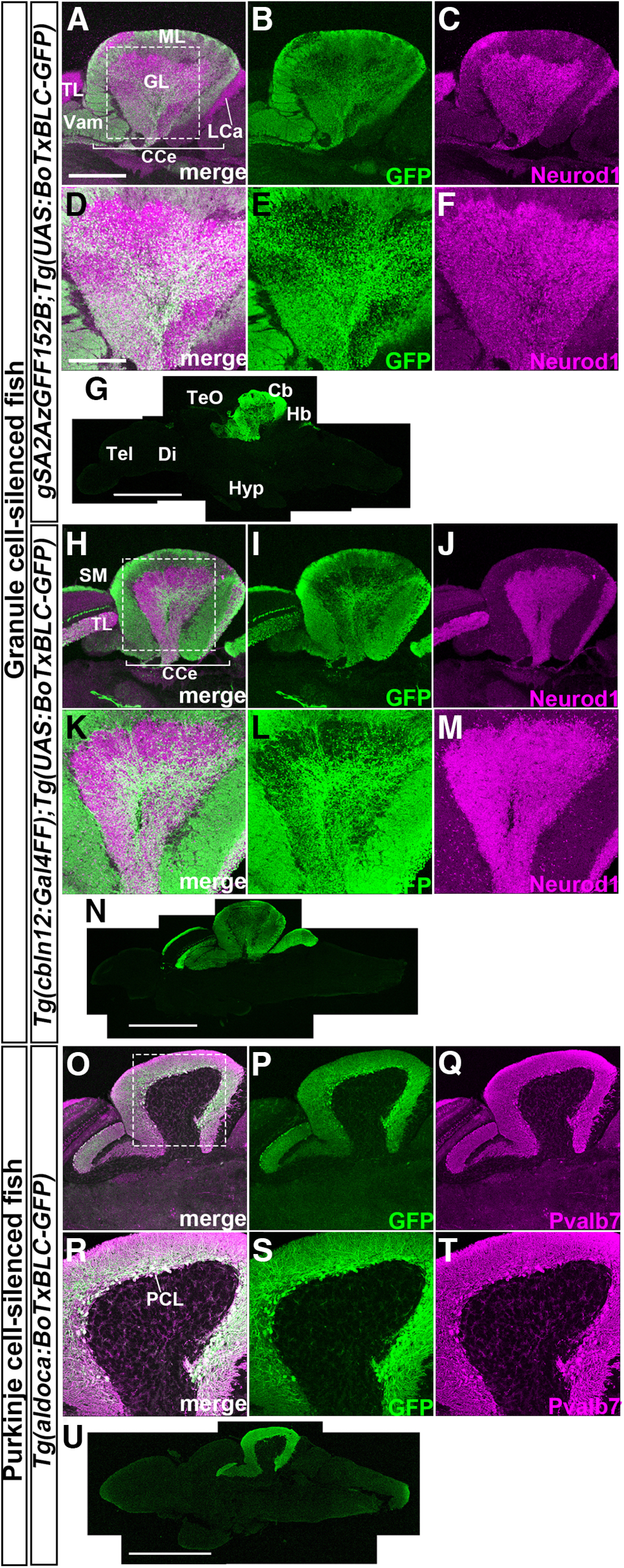
Establishment of Tg fish that express botulinum toxin in GCs or PCs. Sagittal sections of adult *gSA2AzGFF152B;Tg(UAS:BoTxBLC-GFP)* (***A–G***), *Tg(cbln12:Gal4FF);Tg(UAS:BoTxBLC-GFP)* (***H–N***), and *Tg(aldoca:BoTxBLC-GFP)* (***O–U***) brains were stained with anti-GFP (green), and anti-Neurod1, or anti-parvalbumin 7 (Pvalb7, magenta) antibodies. ***A–C***, ***H–J***, ***O–Q***, Cerebellum region. ***D–F***, ***K–M***, ***R–T***, High-magnification views of the boxes in ***A***, ***H***, ***O***. ***G***, ***N***, ***U***, Low-magnification views. Cb, cerebellum; CCe, corpus cerebelli; Di, diencephalon; GL, granular layer; Hb, hindbrain; Hyp, hypothalamus; LCa, lobus caudalis cerebelli; ML, molecular layer; PCL; PC layer; SM, stratum marginale; Tel, telencephalon; TeO, tectum opticum; TL, torus longitudinalis; Vam, medial division of valvula cerebelli. Scale bars: 400 μm (***A***; applies to ***A–C***, ***H–J***, ***O–Q***), 200 μm (***D***; applies to ***D–F***, ***K–M***, ***R–T***), 1 mm (***G***, ***N***, ***U***).

We previously used the Tg line *gSA2AzGFF152B* that expresses a modified version of Gal4-VP16 (Gal4FF) specifically in GCs to study the roles of GCs in classical fear conditioning (Takeuchi et al., 2015; Matsuda et al., 2017; Table 1). In addition to that line, we generated a Tg line, *Tg(cbln12:Gal4FF)*, which expresses Gal4FF in GCs by using an ∼2.0-kbp promoter/enhancer of the *cerebellin12* (*cbln12*) gene, and reportedly drives transgene expression in GCs (Dohaku et al., 2019). We crossed *gSA2AzGFF152B* or *Tg(cbln12:Gal4FF)* with *Tg(UAS:BoTxBLC-GFP)*, which expresses a fusion protein of the botulinum toxin light chain B and green fluorescent protein (BoTxBLC-GFP), which inhibits the synaptic release of neurotransmitters, in a Gal4-dependent manner (Sternberg et al., 2016; Lal et al., 2018), and raised them to adulthood (hereafter referred as to 152B::BoTx and cbln12::BoTx lines; Fig. 1*A–G*,*H–N*). We also generated a Tg line that expresses BoTxBLC-GFP in PCs by using an ∼5.0-kbp promoter/enhancer of the aldolase Ca (*aldoca*) gene, which drives transgene expression specifically in PCs (Tanabe et al., 2010; Takeuchi et al., 2015; Dohaku et al., 2019; *Tg(aldoca:BoTxBLC-GFP)*, hereafter referred as to aldoca:BoTx, Fig. 1*O–U*). We found similar levels of BoTxBLC-GFP expression in the cerebellum of 5-dpf larvae of the same Tg lines. All of these Tg fish did not show any obvious abnormalities, including in swimming behaviors, during development. We dissected the brains from the adults of these Tg fish (more than three months old) after the behavior analyses described below and examined the expression of BoTxBLC-GFP by immunostaining with antibodies against GFP, and a GC marker (Neurod1; Kani et al., 2010) or a PC marker parvalbumin 7 (Pvalb7; Bae et al., 2009). Adult 152B::BoTx fish specifically showed BoTxBLC-GFP expression in GCs, mainly in the CCe (Fig. 1*A–G*), as reported previously for late-stage larvae (Matsuda et al., 2017). Adult cbln12::BoTx fish displayed BoTxBLC-GFP expression in GCs in both the CCe and LCa, as well as in GCs in the torus longitudinalis of the mesencephalon (TL; Fig. 1*H–N*). cbln12::BoTx fish also displayed BoTxBLC-GFP expression in some telencephalic neurons (data not shown) as the *cbln12* promoter was reported to drive transgene expression in telencephalic neurons in addition to GCs (Dohaku et al., 2019). In 152B::BoTx and cbln12::BoTx fish, BoTxBLC-GFP mRNA was transcribed in the somata of GCs while BoTxBLC-GFP protein was transported to the GC axons. Consistent with this, BoTxBLC-GFP was also detected in the molecular layer (ML) of the cerebellum and/or the stratum marginale (SM) of the optic tectum where GC axons were present (Fig. 1*G*,*I*,*N*). A comparison with Neurod1 expression revealed that 60.7% and 11.6% of GCs in the medial and lateral domains, respectively, of the CCe expressed BoTxBLC-GFP in 152B::BoTx fish while only 0.433% of GCs in the LCa expressed BoTxBLC-GFP. In cbln12::BoTx fish, 43.8% and 42.9% of GCs in the medial and lateral regions, respectively, of the CCe, and 49.9% of GCs in the LCa, expressed BoTxBLC-GFP (Table 1). These data suggest that the 152B::BoTx fish preferentially expressed BoTxBLC-GFP in GCs of the medial CCe whereas cbln12B::BoTx expressed it in GCs of both the CCe and LCa. A comparison with Pvalb7 expression indicated that 97.7% of Pvalb7^+^ PCs expressed BoTxBLC-GFP in the aldoca:BoTx line (Table 1). Although we cannot exclude the possibility that BoTx was also expressed in some neurons other than PCs, they are likely to be a minor population. Our data indicate that BoTx was expressed in large numbers of GCs or PCs of the cerebellum of these Tg fish.

### Active avoidance conditioning in adult zebrafish

**Figure 2.**
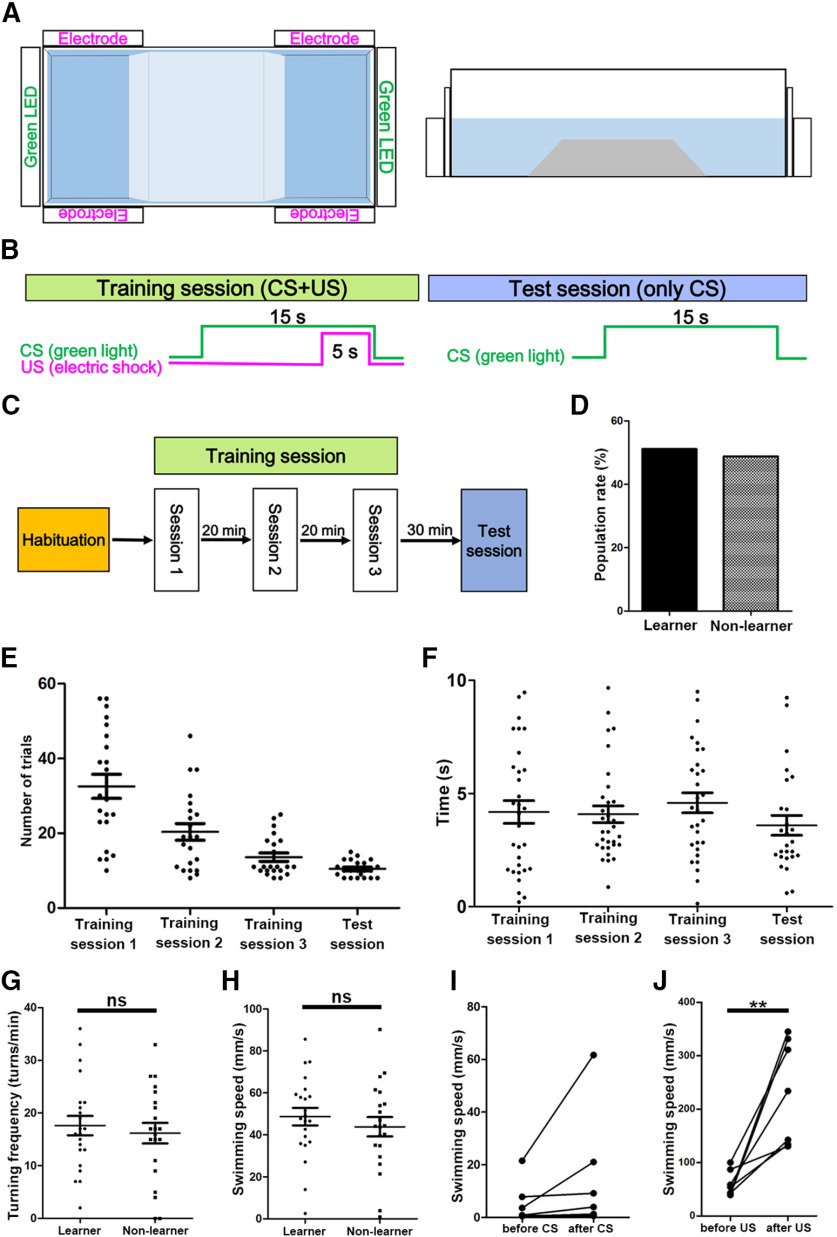
Active avoidance conditioning of wild-type fish. ***A***, Tank used for active avoidance conditioning. A white opaque tank (L41 cm × W17 cm × H12 cm) with transparent walls at both ends, and a trapezoidal wedge (L10–20 cm × W17 cm × H5 cm) in the center, were used. Green LEDs and a pair of electrodes were placed on each side. Top view (left panel) and side view (right panel). ***B***, ***C***, Protocol for active avoidance. In the habituation session, a fish was allowed to swim freely for 20 min in the tank. In the training session, when a fish was located in a side compartment, the LED was turned on for 15 s (CS). If the fish did not escape to the other side after 10 s, an electric shock was administered for 5 s (US) in each trial. When the fish moved before the electric shocks, the trial was successful and was followed by a 30-s interval and the next trial. When fish had eight successful trials among 10 consecutive trials, they were considered to have established active avoidance in the training session, and were subjected to the next trial session. When fish did not establish active avoidance within 60 trials, the training session was terminated. When fish established active avoidance in three consecutive training sessions, they were subjected to the test session. In the test session, only light stimuli with LEDs were administered. When the fish had eight successful trials among 10 consecutive trials in the training session, they were considered to be learners. When fish did not establish active avoidance in the training session or did not succeed in the test session, they were considered to be non-learners. ***D***, Acquisition of active avoidance conditioning in wild-type adult fish. Percentages of learners and non-learners are indicated (*n *=* *43). ***E***, Number of trials when learner fish established active avoidance in the training and test sessions (*n *=* *22). The graph shows averages and SEs of the data. ***F***, Time from CS to escape in each session of learner fish (*n *=* *7). The graph shows averages and SEs of the data. ***G***, ***H***, Swimming behaviors. Turning frequency (turns/min) and swimming speed (mm/s) of learners and non-learners during free swimming (learner; *n *=* *22, non-learner; *n *=* *21). The graph shows averages and SEs of the data (ns indicates non-significance, Welch’s *t* test). ***I***, Freezing response of non-learners. Average swimming speed (mm/s) of seven non-learners before and after the onset of CS in the 44th–53rd trials of training session 1 was calculated. ***J***, Test for responsiveness to electric shocks in wild-type adult fish (*n *=* *7). Swimming speed for 2 s before and after electric shocks was calculated (**p <* *0.01, Welch’s *t* test). ns, not significant.

We employed an assay system for two-way active avoidance learning by using a tank with two compartments (Lal et al., 2018) and a previously published protocol (Aoki et al., 2013). In this system, the two compartments were separated by a fixed trapezoidal wedge allowing fish to move freely from one compartment to the other (Fig. 2*A*). Light exposure to green LEDs and electric shocks were considered as the CS and US, respectively. After habituation for 20 min, when a fish was located in a compartment, it was exposed to CS for 15 s during the training session. When the fish did not escape to the other side after 10 s, a 5-s US was applied (Fig. 2*B*). A 15- to 20-s interval was inserted between trials. When the fish escaped before US during the presentation of CS, it was considered as a successful trial (Movie 1). When the fish did not escape before US, it was considered as a failed trial (Movie 2). We found that no fish (*n *=* *43) escaped to the other side within the first 10 s after the onset of CS during the first trial before receiving any US. Fish that had eight successful trials among 10 consecutive trials were considered to have established active avoidance in the session, that session was terminated, and the fish were subjected to the next session following a 20 min interval (Fig. 2*C*). Each session contained up to 60 trials. The fish that did not establish active avoidance within 60 trials were not subjected to further trials, while those that established active avoidance in three consecutive sessions (sessions 1–3) were subjected to the test session in which only CSs were provided (Fig. 2*C*). When the fish succeeded in eight trials among the 10 consecutive trials within 60 trials, they were considered as learners. Fish that did not establish active avoidance in either training or test sessions were considered as non-learners. A small number of fish that established active avoidance in the training sessions failed in the test session (*n *= 3/43). It is currently unknown why these fish failed after the establishment of active avoidance. In this experimental condition, 51.2% (*n *=* *43) of wild-type adult fish succeeded in establishing active avoidance learning (Fig. 2*D*). Learner fish established active avoidance in 32.5 **±** 3.2, 20.4 **±** 2.2, 13.6 ± 1.1, and 10.5 ± 0.5 trials (average ± SE) in training sessions 1, 2, 3, and the test session, respectively (Fig. 2*E*). Although we could not determine exactly when learners responded to CS to escape, we found that learner fish escaped from the compartment where they received CS at 4.0 **±** 0.63, 4.6 **±** 0.44, 5.0 ± 0.60, and 4.0 ± 0.59 s (average ± SE) after receiving CS in training sessions 1, 2, 3, and the test session, respectively (Fig. 2*F*). We examined average swimming speed and turn frequency during 1 min of free swimming after the habituation session and before the training session. They were not significantly different between learners and non-learners (Fig. 2*G*,*H*). The data indicate that about half of the adult zebrafish could acquire active avoidance conditioning and progressively improved active avoidance. Our findings further suggest that the ability of zebrafish to acquire active avoidance conditioning was not directly related to their ability to swim. Furthermore, non-learner fish did not show a freezing response, i.e., reduction of swimming speed after CS (Fig. 2*I*), indicating that the freezing response was not the cause of the failure to learn.

Movie 1. Active avoidance of a wild-type fish, successful trial. A successful trial of active avoidance of a wild-type learner fish in the 25th trial of training session 1 is shown. The timing of the CS (light exposure with a green LED) and US (electric shock) are indicated. Note that the fish responded to the CS and escaped into the compartment on the other side of the tank. 10.1523/ENEURO.0507-20.2021.video.1

Movie 2. Active avoidance of a wild-type fish, failed trial. A failed trial of active avoidance of a wild-type non-learner fish in the 25th trial of training session 1 is shown. The timing of the CS and US are indicated. Note also that the fish did not escape after the presentation of CS but responded to US. 10.1523/ENEURO.0507-20.2021.video.2

### Inhibition of GC transmission suppresses active avoidance conditioning

**Figure 3.**
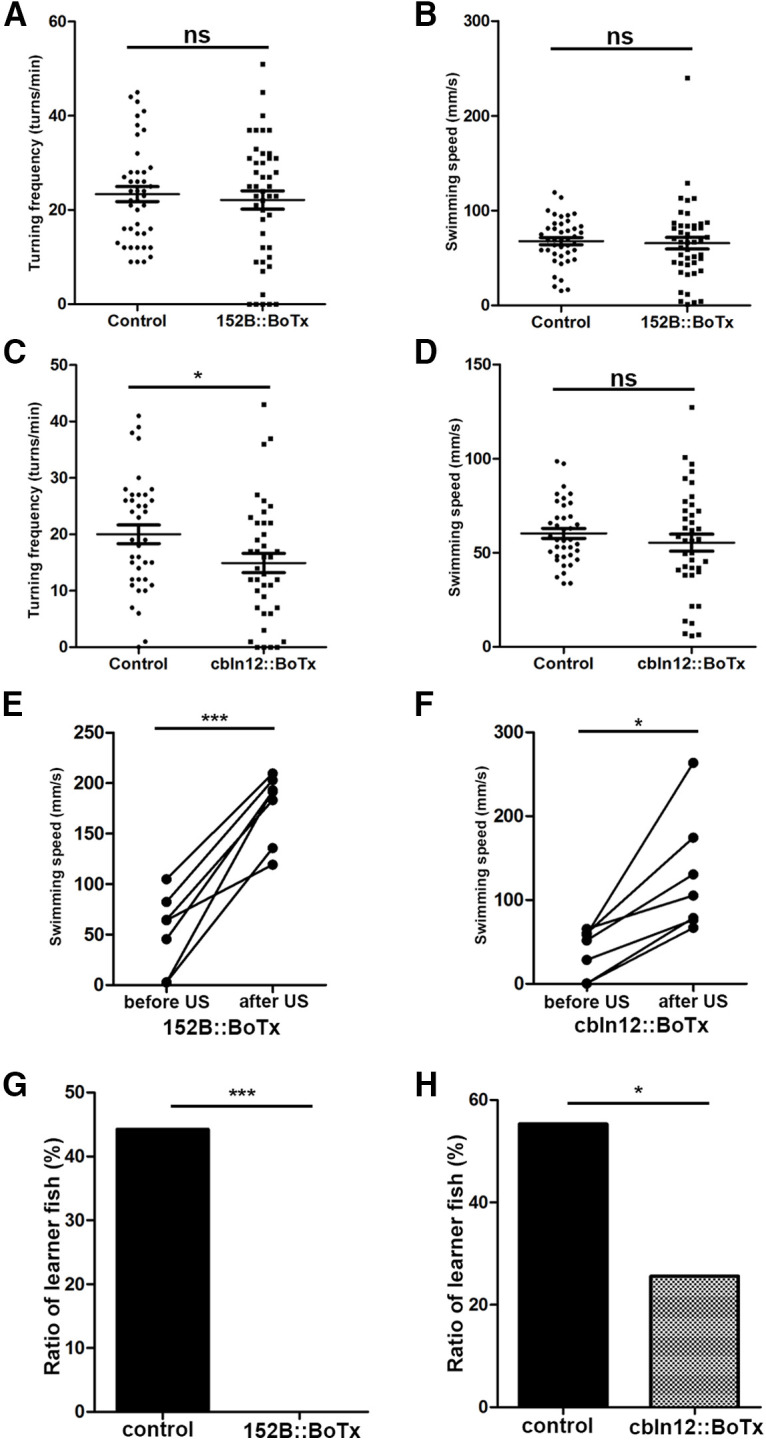
Expression of botulinum toxin in GCs suppresses active avoidance conditioning. ***A***, ***B***, Turning frequency (turns/min) and swimming speed (mm/s) of *gSA2AzGFF152B;Tg(UAS:BoTxBLC-GFP)* (152B::BoTx) and control sibling fish during free swimming (152B::BoTx; *n *=* *47, control; *n *=* *43). The graph shows the averages and SEs of the data (ns indicates non-significance, Welch’s *t* test. ***C***, ***D***, Turning frequency and swimming speed of *Tg(cbln12:Gal4FF);Tg(UAS:BoTxBLC-GFP)* (cbln12::BoTx) fish during free swimming (cbln12::BoTx; *n *=* *39, control; *n *=* *38). The graph shows the averages and SEs of the data (ns indicates non-significance, *p <* *0.05, Welch’s *t* test). ***E***, ***F***, Response to electric shocks in 152B::BoTx (*n *=* *7) and cbln12::BoTx (*n *=* *7) fish. Swimming speed was calculated for 2 s before and after the electric shocks (***p <* *0.001, *p <* *0.05, Welch’s *t* test). ***G***, ***H***, Percentages of active avoidance learners for 152B::BoTx (*n *=* *47) and control sibling fish (*n *=* *43; ***G***), and for cbln12::BoTx (*n *=* *39), and control sibling fish (*n *=* *38; ***H***; ***p <* *0.001, *p <* *0.05, Fisher’s exact test). ns, not significant.

We next analyzed the GC-silenced 152B::BoTx and cbln12::BoTx fish, and compared them with control siblings that did not have Gal4FF and/or BoTxBLC-GFP genes (Fig. 3). Although the cerebellum is involved in some forms of motor control, there was no significant difference in the average swimming speed and turn frequency between 152B::BoTx and control fish (Fig. 3*A*,*B*). Although turn frequency was slightly lower in cbln12::BoTx fish than in control fish (Fig. 3*C*), average swimming speed was not significantly different between cbln12::BoTx and control fish (Fig. 3*D*). Both 152B::BoTx and cbln12::BoTx fish responded to electric shocks as did wild-type fish (Figs. 2*J*, 3*E*,*F*), suggesting that the expression of BoTx did not strongly affect their swimming behaviors or response to US. When 152B::BoTx and control fish were subjected to active avoidance conditioning, 44.2% (*n *=* *43) of control fish were learners while no (*n *=* *45) 152B::BoTx fish were learners (Fig. 3*G*). When cbln12::BoTx and control fish were examined, 55.3% (*n *=* *38) of control fish were learners while 25.6% (*n *=* *39) of cbln12::BoTx fish were learners (Fig. 3*H*). The data indicate that the inhibition of neurotransmitter release in the GCs suppressed active avoidance conditioning. A relatively lower suppression of learner ratio observed in the cbln12::BoTx fish may be because of a relatively lower expression of BoTxBLC-GFP at the single-cell level in cbln12::BoTx fish than in 152B::BoTx fish (Table 1). The data indicate that the BoTx-mediated inhibition of GC transmission suppressed active avoidance conditioning without strongly inhibiting the swimming behaviors or aversive stimuli-dependent escape responses.

### Inhibition of PC transmission suppresses active avoidance conditioning

**Figure 4.**
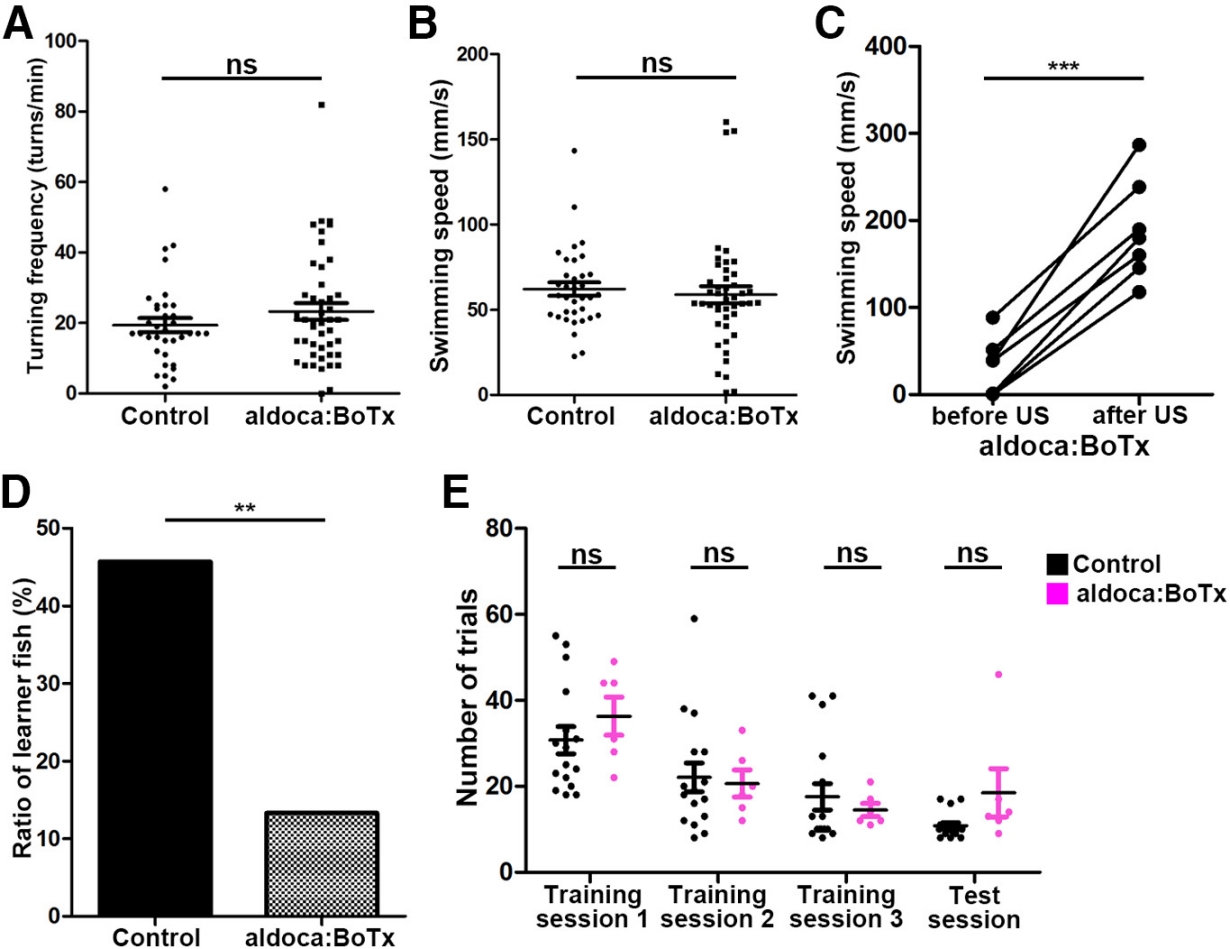
Expression of botulinum toxin in PCs suppresses active avoidance conditioning. ***A***, ***B***, Turning frequency (turns/min) and swimming speed (mm/s) of *Tg(aldoca:BoTxBCL-GFP)* (aldoca:BoTx) and control sibling fish during free swimming (aldoca:BoTx; *n *=* *45, control; *n *=* *35). The graph shows the averages and SEs of the data (ns indicates non-significance, Welch’s *t* test). ***C***, Response to electric shocks in aldoca:BoTx fish (*n *=* *7). Swimming speed was calculated for 2 s before and after the electric shocks (***p <* *0.001, Welch’s *t* test). ***D***, Percentages of active avoidance learners of aldoca:BoTx (*n *=* *45) and control (*n *=* *35) fish (**p <* *0.01, Fisher’s exact test). ***E***, Number of trials required to establish active avoidance conditioning (aldoca:BoTx: *n *=* *6; control: *n *=* *16). The graph shows the averages and SEs of the data (ns indicates non-significance, Welch’s *t* test).

We then analyzed the PC-silenced aldoca:BoTx fish, and compared them with control siblings that did not have the transgene. The average swimming speed and turn frequency were not significantly different between aldoca:BoTx and control fish (Fig. 4*A*,*B*). The aldoca:BoTx fish responded to electric shocks in a manner smilar to wild-type fish (Figs. 2*J*, 4*C*). When fish were subjected to active avoidance conditioning, 45.7% of control sibling fish (*n *=* *35) were learners whereas 13.3% (*n *=* *45) of aldoca:BoTx fish were learners (Fig. 4*D*). Furthermore, although there was no statistically significant difference, the aldoca:BoTx learner fish took more trials than control sibling fish to establish active avoidance in training session 1 and in the test session (Fig. 4*E*). These data indicate that inhibition of neurotransmitter release in PCs suppressed active avoidance conditioning but did not significantly affect conditioning-independent swimming behaviors or aversive stimuli-dependent escape responses.

### Ablation of PCs in adult fish suppresses active avoidance conditioning

**Figure 5.**
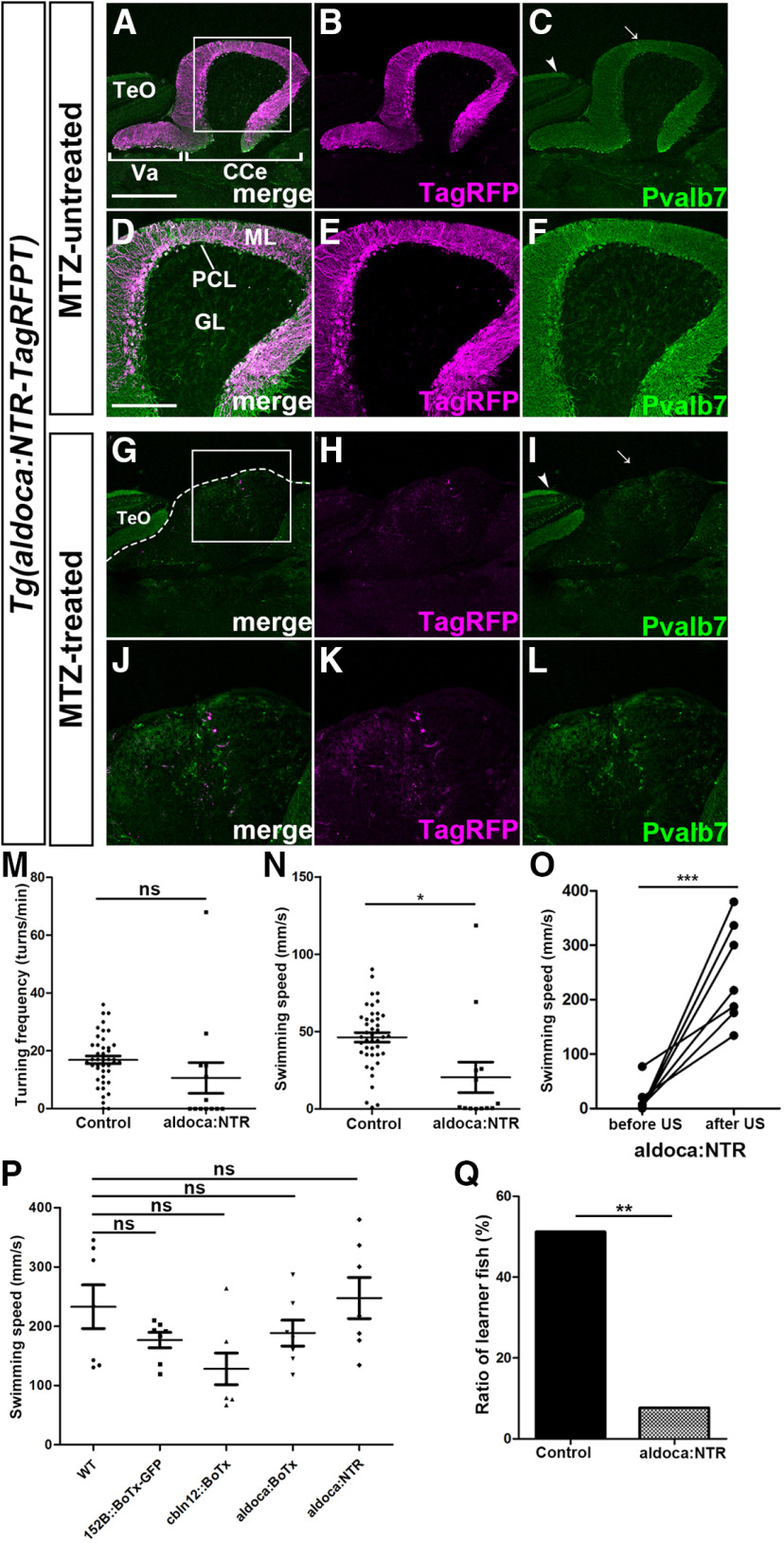
NTR-mediated ablation of PCs in adult fish suppresses active avoidance conditioning. ***A–L***, Ablation of PCs. Adult *Tg(aldoca:NTR-TagRFPT)* fish were treated with MTZ for 18 h (***A–F***) or left untreated (***G–L***). The fish were subjected to behavior assays and subsequent histologic analysis 11 d after MTZ treatment. Sagittal sections were stained with anti-Pvalb7 antibody (green). Expression of NTR-TagRFPT (TagRFP, magenta) is also shown. ***D–F***, ***J–L***, High-magnification views of the boxes in ***A***, ***G***. Arrows and arrow heads indicate Pvalb7-positive dendrites of PCs (in the cerebellum) and Type I neurons (in the optic tectum), respectively. The dotted line in ***G*** indicates the limit of the cerebellum. Note that the Pvalb7 signal in PCs but not in Type I neurons disappeared and no ML was observed in the MTZ-treated fish. ***M***, ***N***, Turning frequency (turns/min) and swimming speed (mm/s) of *Tg(aldoca:NTR-TagRFPT)* (aldoca:NTR) and control fish during free swimming (aldoca:NTR; *n *=* *13, control; *n *=* *43). The graph shows the averages and SEs of the data (ns indicates non-significance, *p <* *0.05, Welch’s *t* test). ***O***, Response to electric shocks in adult aldoca:NTR fish treated with MTZ (*n *=* *7). Swimming speed was calculated for 2 s before and after the electric shocks (***p <* *0.001, Welch’s *t* test). ***P***, Swimming speed for 2 s after US in each strain. The graph shows the averages and SEs of the data (ns indicates non-significance, one-way repeated measures ANOVA with Tukey’s *post hoc* test). ***Q***, Percentage of active avoidance learners of aldoca:NTR (*n *=* *13) and control wild-type (*n *=* *43) fish (**p <* *0.01, Fisher’s exact test). Va, valvula cerebelli. The other abbreviations are described in Figure 1. Scale bars: 400 μm (***A***; applies to ***A–C***, ***G–I***) and 200 μm (***D***; applies to ***D–F***, ***J–L***). ns, not significant.

Since BoTx was expressed in 152B::BoTx, cbln12::BoTx and aldoca:BoTx fish from an early larval stage, rewiring of cerebellar neural circuits might occur and compensate for the deficiency of GC/PC transmission during development. We ablated PCs in adult fish with NTR. We established a Tg line that expresses a fusion protein of modified NTR and TagRFP-T (NTR-TagRFPT; Tabor et al., 2014) in PCs by using the *aldoca* promoter/enhancer [*Tg(aldoca:NTR-TagRFPT)*]. NTR-TagRFPT was expressed from early larval stages to adult stages (Fig. 5*A*,*B*,*D*,*E*). NTR-TagRFPT signals completely overlapped with Pvalb7 signals in the cerebellum but not in the optic tectum where Pvalb7-expressing Type I neurons are present (Fig. 5*C*), indicating that NTR-TagRFPT was specifically expressed in PCs. We found that 11 d after treatment with MTZ, most NTR-TagRFPT-positive and Pvalb7-positive cells disappeared from the cerebellum although Pvalb7-positive Type I neurons remained in the optic tectum while the ML was abrogated (Fig. 5*G–L*), indicating that most PCs were ablated. Turn frequency and swimming speed were reduced in PC-ablated fish compared with control wild-type fish (the difference in turn frequency was not statistically significant; Fig. 5*M*,*N*). However, the PC-ablated fish responded to electric shocks, as did wild-type, 152B::BoTx, cbln12::BoTx, and aldoca:BoTx fish, and swam similarly to wild-type fish after electric shocks (Figs. 2*J*, 5*O*,*P*), suggesting that the acute ablation of PCs slightly affected free swimming but not aversive stimuli-dependent swimming. In active avoidance conditioning, 51.2% of control fish (*n *=* *43) became learners whereas only 7.69% of PC-ablated fish (*n *=* *13) were learners (Fig. 5*Q*), indicating that the ablation of PCs in the adult cerebellum also perturbed active avoidance conditioning.

### Inhibition of GC/PC transmission also suppresses Pavlovian fear conditioning

**Figure 6.**
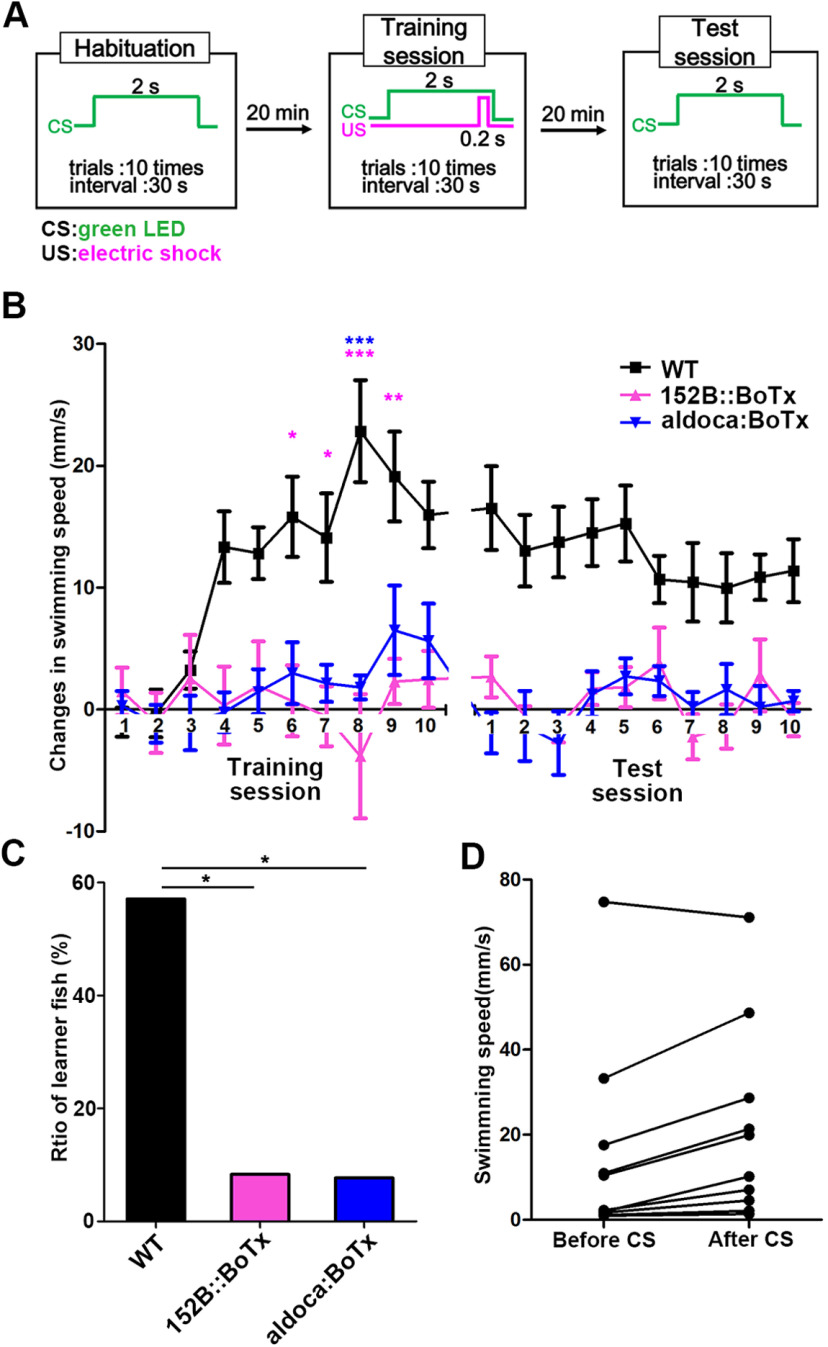
Expression of botulinum toxin in GCs or PCs also perturbs classical conditioning responses. ***A***, Protocol for classical fear conditioning. A compartment on one side of the tank in Figure 2*A* was used. In the habituation session, a light stimulus (CS) was provided for 2 s per trial (10 trials). In the training session, a paired CS and US (0.2-s electric shock given 1.8 s after the onset of CS) was administered in each trial (10 trials). In the test session, CS alone was administered (10 trials). The interval between trials was 30 s, and the interval between sessions was 20 min. ***B***, Changes in swimming speed before and after the CS of wild-type (WT, *n *=* *25), 152B::BoTx (*n *=* *23), and aldoca:BoTx (*n *=* *24) fish. Swimming speed was measured for 1.5 s before and after the CS in each trial, and average changes in swimming speed in the training and test sessions were calculated. The graph shows the averages and SEs of the data (Training session; lines factor: *p *=* *1.483e-07, trials factor: *p *=* *1.189e-07, lines × trials interaction: *p *=* *1.398e-06, two-way repeated measures ANOVA; ***p <* *0.001, **p< 0.01, *p <* *0.05, two-way repeated measures ANOVA with Tukey’s *post hoc* test. Test session; lines factor: *p *=* *1.398e-06, trials factor: *p *=* *0.171, lines × trials interaction: *p *=* *0.3649, two-way repeated measures ANOVA; WT vs 152B::BoTx in test session: *p *<* *1e-22, WT vs aldoca:BoTx in test session: *p *<* *1e-22, 152B::BoTx vs aldoca:BoTx in test session: *p *=* *0.9425, one-way ANOVA with Tukey’s *post hoc* test). ***C***, Percentages of Pavlovian conditioning learners of WT (*n *=* *14), 152B::BoTx (*n *=* *12), and aldoca:BoTx (*n *=* *13) fish (*p <* *0.05, Fisher’s exact test with BH *post hoc* test). ***D***, Pavlovian panic responses during active avoidance conditioning. Data from 10 wild-type fish that were subjected to active avoidance conditioning were used. Since wild-type fish established active avoidance in the 13th trial at the earliest, swimming speed for 1.5 s before and after the CS was measured in each trial from the fourth to the 13th trial. Average speed is plotted in the graph.

Although the inhibition of GCs or PCs suppressed active avoidance conditioning, it is not clear whether it inhibited classical fear conditioning. We conducted Pavlovian fear conditioning in which we determined CS-evoked panic movements by using 152B::BoTx (GC-silenced) and aldoca:BoTx (PC-silenced) fish. In the Pavlovian fear conditioning, light exposure to green LEDs was provided for 2 s as a CS in the habituation session (10 trials), electric shocks were delivered as 0.2-s USs with CSs after the onset of 1.8-s CS in the training session (10 trials), and only CSs were administered in the test session (10 trials; Fig. 6*A*). We assessed the Pavlovian fear conditioned responses by measuring changes in swimming speed for a total of 3 s, i.e., 1.5 s before and after the onset of CS. Wild-type fish progressively increased CS-evoked changes in swimming speed in the training session, and moved quickly after CS in the test session (Fig. 6*B*; Movie 3). The CS-evoked responses were gradually reduced in the test session (Fig. 6*B*). In contrast, the conditioned responses of 152B::BoTx and aldoca:BoTx fish barely improved in the training session (Fig. 6*B*). The conditioned responses in the test session were significantly weaker in 152B::BoTx and aldoca:BoTx fish than in wild-type fish (Fig. 6*B*; Movies 4, 5). We defined “learners” as fish whose conditioned responses were significantly higher in the test session than in the habituation session; 57.1% (*n *=* *14) of wild-type fish were learners whereas a significantly lower number of 152B::BoTx (8.33%, *n *=* *12) and aldoca:BoTx (7.69%, *n *=* *13) fish were leaners (Fig. 6*C*). These data indicate that the inhibition of GC or PC transmission also perturbed the Pavlovian fear conditioning. We further examined Pavlovian fear responses by measuring changes in speed for 1.5 s before and after the onset of CS in active avoidance conditioning; 70% (*n *=* *10) of wild-type fish showed CS-evoked quick movements (Fig. 6*D*), indicating that Pavlovian fear conditioning was also established during active avoidance conditioning.

Movie 3. Pavlovian fear conditioning of a wild-type fish. Behavior of a wild-type learner fish in trial 1 of the test session is shown. The timing of the CS is indicated. Note that the fish moved quickly after the presentation of CS. 10.1523/ENEURO.0507-20.2021.video.3

Movie 4. Pavlovian fear conditioning of a 152B::BoTx fish. Behavior of a 152B::BoTx non-learner fish in trial 1 of the test session is shown. The timing of the CS is indicated. Note that the fish did not move after the presentation of CS. 10.1523/ENEURO.0507-20.2021.video.4

Movie 5. Pavlovian fear conditioning of an aldoca:BoTx fish. Behavior of an aldoca:BoTx non-learner fish in trial 1 of the test session is shown. The timing of the CS is indicated. Note that the fish did not move after the presentation of CS. 10.1523/ENEURO.0507-20.2021.video.5

## Discussion

### Genetic inhibition of cerebellar neurons in zebrafish

The roles of cerebellar neural circuits in fear conditioning have traditionally been studied by inhibiting the cerebellum with physical or laser-induced lesions, or by local administration of an anesthetic drug (Yoshida et al., 2004; Yoshida and Hirano, 2010; Ahrens et al., 2012; Lin et al., 2020). In this study, we inhibited neurotransmitter release from GCs or PCs by expressing BoTx using the promoter/enhancer of GC-expressed or PC-expressed genes (i.e., *cbln12* and *aldoca*) or a Gal4 trap line that drives transgene expression in GCs (Fig. 1). These genetic inhibitions are thought to be reliable, reproducible, and cell-type-specific as the neurotoxin was stably expressed in the cells of interest. The Gal4-UAS system induces transgene expression more strongly than when driven directly by cell-type-specific enhancer/promoters. However, methylation-mediated silencing of UAS-regulated transgenes occurs more as generations progress, resulting in variegated transgene expression (Akitake et al., 2011). Although the *gSA2AzGFF152B* line expresses *Gal4FF* in most GCs of the medial CCe (Takeuchi et al., 2015) and the *cbln12* promoter/enhancer drives transgene expression in all differentiated GCs (Dohaku et al., 2019), the Gal4-dependent expression of BoTxBLC-GFP was mosaic (Fig. 1; Table 1). In contrast, the expression of BoTxBLC-GFP, which was driven directly by the *aldoca* promoter/enhancer (Tanabe et al., 2010), was detected in most, if not all, PCs (Fig. 1; Table 1). Therefore, careful examination of BoTx expression is required to validate results from neuronal inhibition with Gal4-UAS-mediated toxin expression. In this study, even when BoTx was expressed in about half of the GCs of the medial CCe in 152B::BoTx and cbln12::BoTx fish, this suppressed active avoidance conditioning (Fig. 3), suggesting that the establishment of active avoidance conditioning is sensitive to the number of functional GCs in the cerebellum. cbln12::BoTx expressed BoTxBLC-GFP in neurons in the telencephalon and GCs in the TL, in addition to GCs in the cerebellum (Fig. 1). Although the roles of these neurons outside the cerebellum are not necessarily excluded, it is likely that the inhibition of GCs in the cerebellum had a major impact on active avoidance conditioning in both 152B::BoTx and cbln12::BoTx fish. The BoTx-mediated inhibition of PCs also perturbed active avoidance conditioning (Fig. 4). Collectively, BoTx expression driven by both Gal4-UAS or a cell-specific promoter/enhancer successfully inhibited the transmission of GCs and PCs.

PC-silenced fish did not show abnormal locomotion but generated an erratic form of body displacement in some conditions (Chang et al., 2020). However, both GC-silenced and PC-silenced fish did not show conditioning-independent swimming and responded to US in a manner similar to wild-type fish (Figs. 3, 4). Thus, although the BoTx-mediated inhibition of GCs or PCs might affect smooth and/or well-coordinated movements to some extent, its main impact on conditioning is likely to be independent of abnormal swimming behavior.

In this study, BoTx was expressed from early larval stages when GCs and PCs had differentiated. Therefore, physical or functional rewiring of neural circuits might occur and compensate for the deficiency of GC or PC transmission. It might alleviate the effects of inhibition of GCs or PCs. To address this issue, we specifically ablated PCs in the adult cerebellum using the NTR-MTZ system (Fig. 5). Although PC-ablated fish showed some abnormal swimming behaviors, they could respond to US in the same manner as wild-type fish (Figs. 2, 5). Similar to BoTx-expressing fish, PC-ablated fish showed reduced active avoidance conditioning. In contrast, NTR-MTZ-mediated ablation might induce an inflammatory response that exacerbated the cerebellar function, but it did not induce abnormal swimming, such as ataxia or rolling movements. Therefore, the BoTx-mediated inhibition of GCs/PCs and NTR-MTZ-mediated PC ablation were both able to induce defects in cerebellar neural circuits.

### The cerebellum is involved in active avoidance conditioning in zebrafish

Early stage zebrafish larvae acquired operant conditioning in which they learned to turn their tail in the correct direction to obtain relief from an aversive heat stimulus (Lin et al., 2020). In that operant conditioning, cerebellar lesions affected the decision of which direction they would turn their tail (Lin et al., 2020). It is not clear whether the cerebellum is involved in other types of operant conditioning. In this study, we employed two-way active avoidance conditioning with adult zebrafish and genetically inhibited cerebellar neurons. Although the conditioning paradigm and fish ages are different, our findings clearly indicate that the zebrafish cerebellum is involved in active avoidance conditioning.

152B::BoTx fish expressed BoTx mainly in GCs of the medial CCe but only rarely in GCs in the LCa (Fig. 1*C*). This is consistent with previous data in which Gal4FF in *gSA2AzGFF152B* fish drove transgene expression mainly in GCs of the medial CCe in larvae and adult fish (Takeuchi et al., 2015; Matsuda et al., 2017). Since active avoidance conditioning, but not free swimming, was strongly suppressed in 152B::BoTx fish (Fig. 3), GCs in CCe play an important role in active avoidance conditioning but not in free swimming. Previously, it was reported that the inhibition of GC transmission in 152B::BoTx late-stage larvae prolonged fear-conditioned bradycardia responses in which a set of neurons, most likely GCs, in the CCe became activated by the CS and were associated with conditioned bradycardia responses (Matsuda et al., 2017). The same or similar types of neurons might be involved in active avoidance conditioning. As Ca^2+^ and voltage imaging of neurons in freely moving adult zebrafish are still difficult, we were unable to identify conditioning-associated neurons in active avoidance conditioning. Future studies with optical fibers or body-mounted fluorescence sensors that allow for the detection of immediate early gene expression, or virtual-reality conditioning assays, may reveal GCs and PCs that are associated with active avoidance conditioning.

The cerebellar vermis in mammals was reported to be involved in classical fear conditioning, such as autonomic bradycardia and freezing responses (Supple and Leaton, 1990; Supple and Kapp, 1993; Supple et al., 1993; Sacchetti et al., 2002). A previous study (Matsuda et al., 2017), as well as the current study, revealed that GCs in the CCe of the zebrafish cerebellum are involved in conditioned fear responses, including bradycardia and active avoidance. These findings suggest that the CCe of the zebrafish cerebellum has a function similar to that of the mammalian cerebellar vermis.

Cerebellar efferent neurons, which are eurydendroid cells, receive inputs directly from GCs and possibly integrate information of GCs (Harmon et al., 2020). Since BoTx-mediated inhibition of PC transmission or PC ablation in the adult cerebellum perturbed active avoidance conditioning (Figs. 4, 5), the transmission from PCs to eurydendroid cells is required for this conditioning. In goldfish, the activity of major PCs was suppressed whereas that of minor PCs was activated during classical fear conditioning (Yoshida and Kondo, 2012). Long-term depression (LTD) and long-term potentiation (LTP) in GC-PC synapses are involved in cerebellar learning (Ito and Kano, 1982; Salin et al., 1996; Lev-Ram et al., 2002; Sacchetti et al., 2004), although the contribution of LTD or LTP may depend on the type of learning. The inhibition or ablation of PCs might induce an imbalance of PC outputs, resulting in defective active avoidance behaviors. Future analysis of PC activity during conditioning will clarify this issue.

The habenula-raphe circuit and the medial zone of the dorsal telencephalon (Dm), which are thought to correspond to the amygdala in mammals, were involved in active avoidance conditioning in zebrafish (Aoki et al., 2013; Amo et al., 2014; Lal et al., 2018). In mammals, the amygdala plays essential roles in both classical fear conditioning and active avoidance conditioning (Duvarci and Pare, 2014; Herry and Johansen, 2014). Although a functional connection between the habenula and cerebellum was suggested (Lin et al., 2020), future studies on neural circuit connections between the cerebellum and the habenula/Dm are needed and will reveal what kind of information is transmitted to, and integrated in, the cerebellum.

### Roles of the cerebellum in Pavlovian fear and active avoidance conditioning

Previously, BoTx-mediated inhibition of GCs did not perturb but rather prolonged CS-evoked bradycardia responses in zebrafish larvae, suggesting that cerebellar neural circuits control recovery from the fear-conditioned response (Matsuda et al., 2017). In this study, the inhibition of GCs or PCs suppressed CS-evoked Pavlovian panic responses, indicating a positive role of cerebellar neural circuits in the conditioned fear response. Why do the results of cerebellum inhibition differ between the two different classical conditioning paradigms? Although both are forms of classical fear conditioning in which CS-induced passive responses occur after conditioning, the bradycardia response is an autonomic response while the swimming response is a motor response. One possibility is that the two different conditioned responses are controlled by different neural circuits in the cerebellum although they share the same or similar sensory or integration systems, including the habenula nuclei and Dm. A different subpopulation of cerebellar neurons may control autonomic and motor responses. Total ablation of the CCe or anesthetic inhibition of the cerebellum impaired the acquisition of the conditioned bradycardia response in goldfish (Yoshida et al., 2004; Yoshida and Hirano, 2010), supporting the positive role of the entire cerebellum in classical fear conditioning. In 152B::BoTx fish, a specific GC subpopulation that functions in recovery from the conditioned fear response might be suppressed. Alternatively, the same cerebellar neurons may control both autonomic and motor responses in different manners: they regulate the timing of recovery from the freeze response while controlling the decision of the motor response. The elucidation of neurons whose activity was associated with each type of conditioned responses and/or specific inhibition of GC or PC subpopulations in each conditioning paradigm will clarify neural circuits that control the conditioned autonomic and motor responses.

Both active avoidance and Pavlovian fear conditioning involve associative learning of the CS and US, and behavioral decisions based on them. We found that both of them were suppressed by PC or GC inhibition. Pavlovian fear conditioning was also established during active avoidance conditioning (Fig. 6). Although we could not determine the relationship between Pavlovian and active conditioning, it is possible that zebrafish first establish Pavlovian fear conditioning then, simultaneously or subsequently, move directionally to avoid US. In this scenario, the cerebellar neural circuits may control the initial process and possibly the decision-making of active avoidance conditioning. In summary, our findings indicate that the cerebellum plays active roles in active avoidance conditioning and provide a platform for understanding the mechanisms of conditioned fear responses.

## Synthesis

Reviewing Editor: Leonard Maler, University of Ottawa Decisions are customarily a result of the Reviewing Editor and the peer reviewers coming together and discussing their recommendations until a consensus is reached. When revisions are invited, a fact-based synthesis statement explaining their decision and outlining what is needed to prepare a revision will be listed below. The following reviewer(s) agreed to reveal their identity: Haleh Fotowat, Emre Yaksi.

Editorial Comments

Both reviewers found this manuscript interesting and a potentially importand contribution to our understanding of the role of the cerebellum in conditioning. However, there were some fairly serious problems with the Ms that must be addressed.

I have included the detailed reviews so that they can be carefully addressed. Please be sure to provide a clear point by point rebuttal and a redlined Ms in your resubmission.

Reviewer #1

The authors investigated the role of cerebellum in the context of active avoidance conditioning when an unrestrained animal learns to move away from the location of threat in response to a non-threatening conditional stimulus (CS). Although cerebellum is known to play an important role in motor learning, and classical fear conditioning, the role it might play in operant conditioning is not well understood. Previous studies had shown that lesions to cerebellum impairs performance in operant conditioning paradigm, however, the role of specific cell types in this regard was not investigated.

The authors used genetic and chemical ablation techniques to silence cerebellar Purkinje or granule cells and investigated the impact on the behavioral response of adult zebrafish engaged in ‘active avoidance’ and ‘classical fear’ conditioning paradigms. This manuscript provides advances in this field, however, the conclusions made by the authors are limited due to lack of specificity in two of the three transgenic lines. Although the transgenic line that targets Purkinje cells showed great specificity, this was not the case for the transgenic lines that targeted granule cells, partially limiting conclusions that could be made about the role of GCs in fear conditioning.

Major concerns:

More detailed analysis of behavioral data is required in order to support the authors’ claims. Data should be provided on the details of behavioral responses e.g. their relative timing to CS and data from the behavior of both learner and non-learner fish should be provided to support the authors conclusions.

About half of the fish display active avoidance, what did the other half do? Did they generate a freezing response? Did they behave as if nothing happened?

Transgenic lines 152B::BoTx and cbln12::BoTx both show non-specific expression in the brain. The results reported therefore, cannot be attributed to selective ablation of granule cells. This should be discussed in more detail in the results and discussion sections (see one of my specific comments on the discussion section).

It is not clear what is the difference between ‘active avoidance’ vs fear-conditioned ‘involuntary swimming’ response. The only difference between the two conditions as evident in the paper is the time course of stimulus presentation where there is longer delay between CS and US in the classical conditioning paradigm. I assume that an involuntary response would occur at a shorter latency relative to CS but this information is not provided in the paper. Along the same lines, it is unclear why swimming responses observed in the classical conditioning case are ‘involuntary’. This needs to be explained better.

All fish showed a reduction in the percentage of the fish that were ‘learners’. The behavior of Tg learners however was not different from control learners. Was there a difference in expression level between learners and non-learners in the Tg line? Did non-learners have a higher expression level? Was the behavior of non-learners in transgenic lines similar to non-learners in the wild type or do they behave differently? These are all important pieces of information that are currently missing from the paper.

Specific concerns:

Line 43. Explain what you mean by restrained condition here. Is it that the animal is physically restrained and kept in place? Or that the animal has no place to go to avoid the shock?

Line 45. Please define what bradycardia is what exactly happens in the context of classical fear conditioning. Also provide a reference here.

Line 46. The contrasting of behavioral response to voluntary and involuntary just because the animal is restrained or not is incorrect. Again, it is not clear what it is meant here by restrained. But in any case, I expect that ‘involuntary responses’ like autonomic responses still occur in non-restrained animals. Also, restrained animals might still produce ‘voluntary’ responses in the form of attempted escapes.

Line 214. “Adult 152B::BoTx fish specifically showed BoTxBLC-GFP expression in GCs , GCs, mainly in the CCe (Fig. 1A-G)”. From this figure it looks like the expression is really not ‘specific’ to GCs at all. There seems to be a lot of expression outside of the area labeled by Neurod1.

Figure 1A & H. Please mark the CCe and TL region in both panels.

Line 228. Please make a statement regarding specificity to GC/PC vs non-specific expression. The aldoca:BoTx fish showed much more specific expression than the other two lines.

Line 237. What was the percentage of the trials where the fish went to the other side within the first 10 seconds after CS onset on the first trial before having received any US? This is important to know whether CS itself was neutral or not.

Movie 1- legend. Please indicate which point in training this video corresponds to.

Movie 2- legend. Please indicate which point in training this video corresponds to. Also, is this the same fish as in movie 1 or a different fish?

Line 245. Please indicate what were the fish that did not establish active avoidance doing during CS. Was there a change in their swimming speed or turns? Were they freezing?

Line 248. Please provide a plot with the timing of escape relative to the CS and how it changed during training for all Tg lines.

Line 249. Were there fish that established avoidance during training and not test? And these were considered non-learners? Why do you think that might be? Please show training data for this group of ‘non-learners’ as it is shown for the learner fish. Was there a difference in their training data?

Line 251. It is not clear what the ‘number of trials’ signifies in Fig. 2E. Didn’t each session have only 10 trials?

Line 255. When was the 1 min of swimming measured? Was this before or after any training?

Line 277. The statement that although there was no significant difference the cbl fish did worse on session one and test should be removed here. With the same logic you could say that they did better on training session 2 and 3. Also, please provide information about the timing of the escape relative to CS onset for control and tg fish.

Line 280. From Table 1 I read that the expression was higher in the cbl fish and not lower than the 152B fish, same is what I take from Figure 1. Please clarify. Also, please indicate the non-specific expression and how that might play a role in the differences you see.

Line 331. Is the only difference between this protocol and previous one the fact that there is shorter delay between CS and US? And is behavior considered ‘involuntary’ because it occurs at a shorter latency? Again, here it is important to report the timing of the behavior relative to CS in the active avoidance paradigm. Also, in the active avoidance paradigm, do you expect to see the same involuntary response at a short delay after CS? Please provide the same information for the other condition to allow for comparison between the two paradigms or explain why you think the active avoidance paradigm does not include an ‘involuntary’ component. It would be very informative to see whether a similar relationship holds for this measure (change in swim speed 3 s around CS) in the active avoidance conditioning. Especially did learners of active avoidance task also show the involuntary response in the correct direction?

Movies 4 and 5. In both cases the fish doesn’t seem to move much before or after the CS. Were these two fish considered learners or non-learners? Please provide an example videos of learner and non-learner in this case.

Line 373. “Although the roles of these neurons outside the cerebellum are not necessarily excluded, it is likely that the inhibition of GCs in the cerebellum had major impacts on active avoidance conditioning”. Please explain why you believe that the effect you see is likely due to inhibition of GCs and not the unspecific effects?

Line 405. What do you mean by ‘two-way’ avoidance conditioning? This is the first time the term is used in the paper. Please clarify. How is the operant conditioning paradigm here different from the one in Lin et al?

Line 451. This sentence is very hard to follow. Do you mean bradycardia signifies recovery from a fear response? And inhibition of GCs removes the suppression of bradycardia response? Please clarify this sentence.

Line 461. What do you mean by ‘autonomic freeze’? Freezing response could very well be voluntary and it is a motor response.

Line 472. Did you ever observe freezing response in your experiments? If so were the freezing fish considered non-learners? Please provide this information in the results section.

Reviewer #2

In this manuscript the authors investigated the role of cerebellar purkinje cells (PCs) and granule cells (GCs) in active avoidance and classical fear learning tasks, using adult zebrafish. To achieve this, the authors first developed 2 new transgenic zebrafish lines to label and silence PCs and GCs. Later they adopted investigated the role of silencing/ablating both of these cell types during learning behavior. The authors showed that perturbing both of these cerebellar cell types reduce animals ability to learn during active avoidance and classical fear learning, without a major impact on animals natural swimming behaviors..

1) In general, it would help a lot to relate these main findings to what we know about the connection between cerebellar circuits and active avoidance learning and classical fear learning in mammals and in other animal models. For example, I wondered if there is a specific sub division of cerebellum responsible for this task. I do not ask for experimentation but some additional intro and discussion might help

2) It appears from the comparison of graphs in Figure 2H, and 3 E,F that wild type animals have a stronger response to US delivery. Can the authors please compare if the US responses of GC and PC ablated/silenced animals are significantly different from wild type animals. Does this mean that GC ablated animals animals can perceive the US less efficiently and hence this might contribute to reduced learning performance ? I do not ask for new experimentation but just an additional comparison and a short additional discussion on what these results might mean, if there is a difference in swim response to US. I think that this comparison above is especially important this the authors argue that silencing GCs doesnt alter aversive stimuli dependent escape response, in line 283

3) where else in the brain is silenced using 152B:BoTx and cbln12:BoTx fish lines ? can the authors please clarify and highlight this in the text and perhaps add a supplementary image about this expression beyond cerebellum (if there is any) ??? I wasn’t sure from the discussion line 375 if these lines are only expression PCs and GCs or also elsewhere in the brain

## Author Response

Editorial Comments

Both reviewers found this manuscript interesting and a potentially important contribution to our understanding of the role of the cerebellum in conditioning. However, there were some fairly serious problems with the Ms that must be addressed. I have included the detailed reviews so that they can be carefully addressed. Please be sure to provide a clear point by point rebuttal and a redlined Ms in your resubmission.

> We are grateful to the reviewers for their positive comments and suggestions regarding the significance and novelty of our findings. We have carefully considered all of the issues that were raised by the reviewers and carried out additional experiments to address each of them, where necessary. A point-by-point detailed response has been provided below for each of the reviewers’ comments.

Reviewer #1

The authors investigated the role of cerebellum in the context of active avoidance conditioning when an unrestrained animal learns to move away from the location of threat in response to a non-threatening conditional stimulus (CS). Although cerebellum is known to play an important role in motor learning, and classical fear conditioning, the role it might play in operant conditioning is not well understood. Previous studies had shown that lesions to cerebellum impairs performance in operant conditioning paradigm, however, the role of specific cell types in this regard was not investigated.

The authors used genetic and chemical ablation techniques to silence cerebellar Purkinje or granule cells and investigated the impact on the behavioral response of adult zebrafish engaged in ‘active avoidance’ and ‘classical fear’ conditioning paradigms. This manuscript provides advances in this field, however, the conclusions made by the authors are limited due to lack of specificity in two of the three transgenic lines. Although the transgenic line that targets Purkinje cells showed great specificity, this was not the case for the transgenic lines that targeted granule cells, partially limiting conclusions that could be made about the role of GCs in fear conditioning.

> The reviewer appears to be concerned about the specificity of transgenic lines that targeted granule cells (GCs), compared with those that targeted Purkinje cells. The reviewer might consider the following: BoTxBLC-GFP was expressed not only in the granular layer (GL) but also in the molecular layer (ML) of the cerebellum and the stratum marginale (SM) of the optic tectum in 152B::BoTx and cbln12::BoTx (Fig. 1). As published previously (Takeuchi et al., 2017; Dohaku et al., 2019), the gSA2AzGFF152B and cbln12 promoter drive expression of transgenes specifically in GCs of the cerebellum and the torus longitudinalis (TL) of the mesencephalon. In 152B::BoTx and cbln12::BoTx fish, while the BoTxBLC-GFP gene was expressed in the somata of GCs, the BoTxBLC-GFP protein was transported to axons of GCs that were located in the ML of the cerebellum and the SM of the optic tectum. Although BoTxBLC-GFP was observed in neurons in the telencephalon in cbln12::BoTx fish, BoTxBLC-GFP expression was specific to GCs within the mesencephalon and the cerebellum. GC-specific expression of BoTxBLC-GFP in 152B::BoTx fish was reported previously (Matsuda et al., 2017). To avoid any misunderstandings, we have described “In 152B::BoTx and cbln12::BoTx fish, BoTxBLC-GFP mRNA was transcribed in the somata of GCs while BoTxBLC-GFP protein was transported to the GC axons. Consistent with this, BoTxBLC-GFP was also detected in the molecular layer (ML) of the cerebellum and/or the stratum marginale (SM) of the optic tectum where GC axons were present (Fig. 1G, I, N)” in lines 226-231. We have also labeled the SM in Fig. 1.

Major concerns:

More detailed analysis of behavioral data is required in order to support the authors’ claims. Data should be provided on the details of behavioral responses e.g. their relative timing to CS and data from the behavior of both learner and non-learner fish should be provided to support the authors conclusions.

> In active avoidance, some fish were actively swimming while others were swimming slowly or did not move during the onset of CS. There were various CS-evoked responses of learners such as increased swimming speed and making turns. It is difficult to determine precisely when fish responded to CS. We examined responses when fish escaped from the compartment where they received CS. The average escape time was around 4 s. However, this does not necessary imply that they responded at that moment. We described this as follows: “Although we could not determine exactly when learners responded to CS, we found that learner fish escaped from the compartment where they received CS at 4.0 {plus minus} 0.63, 4.6 {plus minus} 0.44, 5.0 {plus minus} 0.60, and 4.0 {plus minus} 0.59 s (average {plus minus} SE) after receiving CS in training sessions 1, 2, 3, and the test session, respectively” in lines 272-276 and show the data in Fig. 2F of the revised manuscript.

About half of the fish display active avoidance, what did the other half do? Did they generate a freezing response? Did they behave as if nothing happened?

> We examined the freezing response of wild-type non-learner fish during active avoidance. We compared swimming speed of seven non-learner fish for 7 s before and after the onset of CS in the last ten trials. We did not observe a freezing response (reduced swimming speed after training) of those fish. The data suggest that the freezing response was not the cause of their failure to learn. We have shown the data in Fig. 2I and described this issue in lines 282-284 in the revised manuscript.

Transgenic lines 152B::BoTx and cbln12::BoTx both show non-specific expression in the brain. The results reported therefore, cannot be attributed to selective ablation of granule cells. This should be discussed in more detail in the results and discussion sections (see one of my specific comments on the discussion section).

> As described above, the gSA2AzGFF152B and cbln12 promoter drives expression of transgenes specifically in granule cells of the cerebellum and the torus longitudinalis (TL) of the mesencephalon (Takeuchi et al., 2017; Dohaku et al., 2019). In 152B::BoTx and cbln12::BoTx fish, the BoTxBLC-GFP gene was expressed in the somata of GCs, while BoTxBLC-GFP protein was transported to axons of GCs that were located in the ML of the cerebellum and the SM of the optic tectum. Although BoTxBLC-GFP was expressed in some telencephalic neurons in cbln12::BoTx fish, BoTxBLC-GFP expression was specific to GCs in the mesencephalon and the cerebellum. GC-specific expression of BoTxBLC-GFP in 152B::BoTx fish was previously reported (Matsuda et al., 2017). To avoid any misunderstanding, we have described “In 152B::BoTx and cbln12::BoTx fish, BoTxBLC-GFP mRNA was transcribed in the somata of GCs while BoTxBLC-GFP protein was transported to the GC axons. Consistent with this, BoTxBLC-GFP was also detected in the molecular layer (ML) of the cerebellum and/or the stratum marginale (SM) of the optic tectum where GC axons were present (Fig. 1G, I, N)” in lines 226-231. We have also labeled the SM in Fig. 1.

It is not clear what is the difference between ‘active avoidance’ vs fear-conditioned ‘involuntary swimming’ response. The only difference between the two conditions as evident in the paper is the time course of stimulus presentation where there is longer delay between CS and US in the classical conditioning paradigm. I assume that an involuntary response would occur at a shorter latency relative to CS but this information is not provided in the paper. Along the same lines, it is unclear why swimming responses observed in the classical conditioning case are ‘involuntary’. This needs to be explained better.

> We agree with the reviewer. Active avoidance is a voluntary response as fish decide to move directionally to escape. However, it is not certain whether or not fear responses with a short latency (Pavlovian panic responses) that we analyzed in Fig. 6 are voluntary. We decided not to use the term ‘involuntary conditioned fear response’. To explain this issue, we have described “Although the inhibition of GCs or PCs suppressed active avoidance conditioning, it is not clear whether it inhibited classical fear conditioning. We conducted Pavlovian fear conditioning in which we determined CS-evoked panic movements by using 152B::BoTx (GC-silenced) and aldoca:BoTx (PC-silenced) fish” in lines 349-351 of the revised manuscript.

All fish showed a reduction in the percentage of the fish that were ‘learners’. The behavior of Tg learners however was not different from control learners. Was there a difference in expression level between learners and non-learners in the Tg line?

Did non-learners have a higher expression level?

> We examined the expression of BoTxBLC-GFP in three individuals of 152B::BoTx, cbln12::BoTx, and aldoca:BoTx fish (Fig. 1 and Table 1). The expression of BoTxBLC-GFP was not very different among the three fish of the same Tg line. Furthermore, we examined BoTxBLC-GFP in the cerebellum of these Tg larvae (5-dpf larvae). We did not observe any difference among the larvae of the same Tg line. There was not much difference in BoTxBLC-GFP expression in individual fish of the same Tg lines. Therefore, the ability of zebrafish to acquire active avoidance conditioning might not be related to expression levels of BoTx. To conclude this issue, we need to generate a large number of Tg fish, and to examine BoTxBLC-GFP expression after conditioning, and perform statistical analysis. We were not able to complete this within the limited revision time period. We have explained similar levels of expression of BoTxBLC-GFP in the cerebellum of the same Tg lines in lines 212-214.

Was the behavior of non-learners in transgenic lines similar to non-learners in the wild type or do they behave differently? These are all important pieces of information that are currently missing from the paper.

> Non-leaners of wild-type and transgenic fish did not show specific behaviors, such as freezing responses (described above). There were no significant differences in swimming behaviors (turning frequency and swimming speed) between wild-type, 152B::BoTx, and aldoca:BoTx non-learners. As we have no additional information, we did not describe or discuss this issue further.

Specific concerns:

Line 43. Explain what you mean by restrained condition here. Is it that the animal is physically restrained and kept in place? Or that the animal has no place to go to avoid the shock?

> We have deleted the description related to “restrained condition” since even if an animal is physically restrained, it could show both passive and active conditioned responses, as the reviewer mentioned. We have changed this sentence to “It shows two types of fear responses, ‘passive’ and ‘active’, upon exposure to a CS. ‘Passive’ responses are known as “classical fear conditioning” or “Pavlovian fear conditioning”.

Line 45. Please define what bradycardia is what exactly happens in the context of classical fear conditioning. Also provide a reference here.

> We have changed this sentence to “This includes motor responses, including freezing behavior and panic movements (Sacchetti et al., 2002; Agetsuma et al., 2010; Amo et al., 2014; Lal et al., 2018), and autonomic reactions such as CS-evoked bradycardia responses (Supple and Leaton, 1990; Supple and Kapp, 1993; Supple et al., 1993; Yoshida and Hirano, 2010; Kotajima et al., 2014; Matsuda et al., 2017).

Line 46. The contrasting of behavioral response to voluntary and involuntary just because the animal is restrained or not is incorrect. Again, it is not clear what it is meant here by restrained. But in any case, I expect that ‘involuntary responses’ like autonomic responses still occur in non-restrained animals. Also, restrained animals might still produce ‘voluntary’ responses in the form of attempted escapes.

> We deleted the description related to “restrained condition”. We changed the sentence to “It shows two types of fear responses, ‘passive’ and ‘active’, upon exposure to a CS....... It also shows ‘active’ responses that result in the avoidance of noxious stimuli”.

Line 214. “Adult 152B::BoTx fish specifically showed BoTxBLC-GFP expression in GCs , GCs, mainly in the CCe (Fig. 1A-G)”. From this figure it looks like the expression is really not ‘specific’ to GCs at all. There seems to be a lot of expression outside of the area labeled by Neurod1.

> As we explained above, the BoTxBLC-GFP gene was expressed in GCs while translated BoTxBLC-GFP protein was transported to axons that are located in the ML of the cerebellum and the SM of the optic tectum. BoTxBLC-GFP expression was specific to GCs as described previously (Matsuda et al., 2017).

Figure 1A & H. Please mark the CCe and TL region in both panels.

> We have marked the CCe and TL in Fig. 1A and H.

Line 228. Please make a statement regarding specificity to GC/PC vs non-specific expression. The aldoca:BoTx fish showed much more specific expression than the other two lines.

> The BoTxBLC-GFP expression in 152B::BoTx and cbln12::BoTx fish was specific to GCs, just as the BoTxBLC-GFP expression in aldoca:BoTx fish was specific to PCs. We do not think that we need to make a statement regarding specificity to GC/PC vs non-specific expression.

Line 237. What was the percentage of the trials where the fish went to the other side within the first 10 seconds after CS onset on the first trial before having received any US? This is important to know whether CS itself was neutral or not.

> We examined what percentage of the fish escaped to the other side within the first 10 sec after CS onset and before receiving US during the first trials. We found that no fish moved to the other side (n=43). We found no or a few Tg fish that escaped to the other side during the first trial (152B::BoTx, n=4/47; cbln12::BoTx, n=0/39; aldoca:BoTx, n=2/45; aldoca:NTR, n=0/13). We described the data from wild-type fish “We found that no fish (n = 43) escaped to the other side within the first 10 s after the onset of CS during the first trial before receiving any US” in line 255-257.

Movie 1- legend. Please indicate which point in training this video corresponds to.

Movie 2- legend. Please indicate which point in training this video corresponds to. Also, is this the same fish as in movie 1 or a different fish?

> Movie 1 corresponds to a successful trial of wild-type learner fish (25th trial of the training session 1). Movie 2 corresponds to a failed trial of wild-type non-learner fish (25th trial of the training session 1). We have described this information in the legend of Movies 1 and 2.

Line 245. Please indicate what were the fish that did not establish active avoidance doing during CS. Was there a change in their swimming speed or turns? Were they freezing?

> As described above, we did not observe any freezing response (reduction of swimming speed after training) of non-learner fish (Fig. 2I). We described this issue in lines 282-284.

Line 248. Please provide a plot with the timing of escape relative to the CS and how it changed during training for all Tg lines.

> As described above, it is difficult to determine precisely when the fish responded to CS. We examined when the fish escaped from the compartment upon CS. The average escape time was around 4 s. However, this does not necessarily mean that they responded in that period. We have described “Although we could not determine when exactly learners responded to CS, we found that learner fish escaped from the compartment where they received CS at 4.0 {plus minus} 0.63, 4.6 {plus minus} 0.44, 5.0 {plus minus} 0.60, and 4.0 {plus minus} 0.59 s (average {plus minus} SE) after receiving CS in training sessions 1, 2, 3, and the test session, respectively” in lines 272-276 and show the data in Fig. 2F in the revised manuscript.

Line 249. Were there fish that established avoidance during training and not test? And these were considered non-learners? Why do you think that might be?

> A small number of fish that established active avoidance in the training sessions failed in the test session (n = 3/43). It is possible that the fish became fatigued during training sessions. However, it is currently unknown why these fish failed after the establishment of active avoidance. We have described this issue in lines 266-268.

Please show training data for this group of ‘non-learners’ as it is shown for the learner fish. Was there a difference in their training data?

> The three fish showed a similar number of trials required to establish active avoidance conditioning (reference Fig. 1). However, since there were only three fish, we cannot conclude the reason why those fish established active avoidance in training but not during test sessions. We have decided not to show the data in the revised manuscript.

Line 251. It is not clear what the ‘number of trials’ signifies in Fig. 2E. Didn’t each session have only 10 trials?

> When fish had passed eight successful trials from among the first 10 trials, we did not examine them after the 10th trial in that session. We changed the sentence to “Fish that had eight successful trials among 10 consecutive trials were considered to have established active avoidance in the session, that session was terminated, and the fish were subjected to the next session following a 20-min interval (Fig. 2C)”. If the reviewer feels that we need to explain more, we will gladly add more details.

Line 255. When was the 1 min of swimming measured? Was this before or after any training?

> We measured swimming before the training sessions. We changed the sentence to “We examined average swimming speed and turn frequency during 1 min of free swimming after the habituation session and before the training session. They were no significantly different between learners and non-learners (Fig. 2G, H)” in lines 276-279.

Line 277. The statement that although there was no significant difference the cbl fish did worse on session one and test should be removed here. With the same logic you could say that they did better on training session 2 and 3. Also, please provide information about the timing of the escape relative to CS onset for control and tg fish.

> We have removed the statement and Figure 3I after examining the reviewer’s suggestion. As described above, it is difficult to examine the timing of escape. Although we analyzed when wild-type learner fish moved from the compartment where they received CS in training sessions 1, 2, 3, and the test session (Fig. 2F), we did not analyze this for cbln12::BoTx learner fish because the number of cbln12::BoTx learner fish was too small to make any reliable conclusion.

Line 280. From Table 1 I read that the expression was higher in the cbl fish and not lower than the 152B fish, same is what I take from Figure 1. Please clarify. Also, please indicate the non-specific expression and how that might play a role in the differences you see.

> It is difficult to estimate how much BoTxBLC-GFP was expressed in each granule cell because it was localized in granule cell axons. We speculate that there is a lower expression of BoTxBLC-GFP at the single-cell level in cbln12::BoTx fish than in 152B::BoTx fish. We added “at the single cell level” in this sentence (line 303). As described above, we believe that BoTxBLC-GFP expression was specific to GCs in the mesencephalic tectum and the cerebellum in 152B::BoTx fish.

Line 331. Is the only difference between this protocol and previous one the fact that there is shorter delay between CS and US? And is behavior considered ‘involuntary’ because it occurs at a shorter latency? Again, here it is important to report the timing of the behavior relative to CS in the active avoidance paradigm. Also, in the active avoidance paradigm, do you expect to see the same involuntary response at a short delay after CS? Please provide the same information for the other condition to allow for comparison between the two paradigms or explain why you think the active avoidance paradigm does not include an ‘involuntary’ component. It would be very informative to see whether a similar relationship holds for this measure (change in swim speed 3 s around CS) in the active avoidance conditioning. Especially did learners of active avoidance task also show the involuntary response in the correct direction?

> We examined the conditioned responses by measuring changes in swimming speed for 3 s in the active avoidance conditioning (from the 4th to 13th trial) as we did for Pavlovian fear conditioning. We found that 70 % (n = 10) of wild-type fish showed conditioned responses. However, it was difficult to determine whether Pavlovian conditioning was established first and then active avoidance took place because the two assays were quite different: Pavlovian learning was judged based on 10 specific trials, while active avoidance was judged on the success rates of 60 trials. We have shown the data in Fig. 6D and described it as follows: “We further examined Pavlovian fear responses by measuring changes in speed for 1.5 s before and after the onset of CS in active avoidance conditioning. 70% (n = 10) of wild-type fish showed CS-evoked quick movements (Fig. 6D), indicating that Pavlovian fear conditioning was also established during active avoidance conditioning”. We discussed the possibility that zebrafish first establish the Pavlovian fear conditioning then, simultaneously or subsequently, moved directionally to avoid US in lines 369-373 in the Discussion.

Movies 4 and 5. In both cases the fish doesn’t seem to move much before or after the CS. Were these two fish considered learners or non-learners? Please provide an example videos of learner and non-learner in this case.

> We have shown the behavior of 152B::BoTx (Movie 4) and aldoca:BoTx ‘non-learner’ fish (Movie 5). We do not think that we need to show CS-evoked movements of transgenic learner fish. Since we showed the statistical data in Fig. 6, we would like to show typical data in the videos. We have revised the legends of Movies 4 and 5.

Line 373. “Although the roles of these neurons outside the cerebellum are not necessarily excluded, it is likely that the inhibition of GCs in the cerebellum had major impacts on active avoidance conditioning”. Please explain why you believe that the effect you see is likely due to inhibition of GCs and not the unspecific effects?

> We repeatedly explained the specific expression of BoTxBLC-GFP in GCs in 152B::BoTx fish. It was also reported previously (Matsuda et al., 2017). Although some expression of BoTxBLC-GFP was observed in telencephalic neurons other than GCs, we intended to discuss this issue carefully, as well as other possibilities.

Line 405. What do you mean by ‘two-way’ avoidance conditioning? This is the first time the term is used in the paper. Please clarify. How is the operant conditioning paradigm here different from the one in Lin et al?

> Two-way active avoidance conditioning is commonly used to examine active avoidance in mice and was adapted for zebrafish (Aoki et al., 2013; Lai et al., 2018). In this system, the two compartments are separated by a space allowing fish to move from one compartment to the other. After a fish establishes active avoidance conditioning, a CS from the left causes the fish to escape from left to right, and a CS from the right causes the fish to escape from right to left. We have explained this method in lines 245-248 and in the materials and methods. We have also briefly explained the operant conditioning performed by Lin et al. (2020) in lines 95-96. If the reviewer thinks we need to explain more about the method used by Lin et al., we will follow that suggestion.

Line 451. This sentence is very hard to follow. Do you mean bradycardia signifies recovery from a fear response? And inhibition of GCs removes the suppression of bradycardia response? Please clarify this sentence.

> The sentence was incorrect. We have corrected this sentence to “Previously, BoTx-mediated inhibition of GCs did not perturb but rather prolonged CS-evoked bradycardia responses in zebrafish larvae, suggesting that cerebellar neural circuits control recovery from the fear-conditioned response (Matsuda et al., 2017)”.

Line 461. What do you mean by ‘autonomic freeze’? Freezing response could very well be voluntary and it is a motor response.

> We think that the word ‘autonomic freeze’ was misleading. We have described the bradycardia response as an autonomic response and the swimming response as a motor response in this paragraph of the Discussion.

Line 472. Did you ever observe freezing response in your experiments? If so were the freezing fish considered non-learners? Please provide this information in the results section.

> As described above, we have examined the freezing response of non-learner fish. We did not observe a freezing response (reduction of swimming speed after training) of non-learner fish (Fig. 2I). We have described this issue in lines 282-284.

Reviewer #2

In this manuscript the authors investigated the role of cerebellar purkinje cells (PCs) and granule cells (GCs) in active avoidance and classical fear learning tasks, using adult zebrafish. To achieve this, the authors first developed 2 new transgenic zebrafish lines to label and silence PCs and GCs. Later they adopted investigated the role of silencing/ablating both of these cell types during learning behavior. The authors showed that perturbing both of these cerebellar cell types reduce animal’s ability to learn during active avoidance and classical fear learning, without a major impact on animals natural swimming behaviors.

1) In general, it would help a lot to relate these main findings to what we know about the connection between cerebellar circuits and active avoidance learning and classical fear learning in mammals and in other animal models. For example, I wondered if there is a specific sub division of cerebellum responsible for this task. I do not ask for experimentation but some additional intro and discussion might help

> In mammals, inhibition of the cerebellar vermis resulted in suppression of classical fear conditioning, such as autonomic bradycardia and freezing responses (Supple and Leaton, 1990; Supple and Kapp, 1993, Supple et al., 1993; Sacchetti et al., 2002). We have explained this issue in the Introduction. Furthermore, we discussed the similarity between the CCe of the zebrafish cerebellum and the vermis of the mammalian cerebellum in lines 456-462 of the revised manuscript.

2) It appears from the comparison of graphs in Figure 2H, and 3 E, F that wild type animals have a stronger response to US delivery. Can the authors please compare if the US responses of GC and PC ablated/silenced animals are significantly different from wild type animals? Does this mean that GC ablated animals can perceive the US less efficiently and hence this might contribute to reduced learning performance? I do not ask for new experimentation but just an additional comparison and a short additional discussion on what these results might mean, if there is a difference in swim response to US. I think that this comparison above is especially important this the authors argue that silencing GCs doesn’t alter aversive stimuli dependent escape response, in line 283

> We compared responses to US in wild-type, 152B::BoTx, cbln12::BoTx, and aldoca:NTR fish by calculating the change in swimming speed before and after US. There were no significant differences between wild-type and transgenic fish. We have shown the data in Fig. 5P and described it as “the PC-ablated fish responded to electric shocks as did wild-type, 152B::BoTx, cbln12::BoTx, and aldoca:BoTx fish, and swam similarly to wild-type fish after electric shocks (Fig. 2J, 5O-P)” in lines 340-342.

3) where else in the brain is silenced using 152B::BoTx and cbln12::BoTx fish lines ? can the authors please clarify and highlight this in the text and perhaps add a supplementary image about this expression beyond cerebellum (if there is any) ??? I wasn’t sure from the discussion line 375 if these lines are only expression PCs and GCs or also elsewhere in the brain

> 152B::BoTx and cbln12:BoTx fish expressed BoTxBLC-GFP in GCs in the cerebellum and the torus longitudinalis (TL). cbln12:BoTx fish also displayed BoTxBLC-GFP expression in telencephalic neurons as the cbln12 promoter was reported to drive transgene expression in telencephalic neurons in addition to GCs (Dohaku et al., 2019). We confirmed the BoTxBLC-GFP expression in telencephalic neurons in Tg larvae. It is difficult to show it in sections because there were very few BoTxBLC-GFP-expressing neurons. We have decided to focus this issue as a description only (lines 221-226). As we cannot exclude the expression of BoTxBLC-GFP in a minor cell population, we have carefully described this issue in lines 239-241.
